# Differential in vivo roles of Mpl cytoplasmic tyrosine residues in murine hematopoiesis and myeloproliferative disease

**DOI:** 10.1038/s41375-024-02219-5

**Published:** 2024-03-15

**Authors:** Kira Behrens, Maria Kauppi, Elizabeth M. Viney, Andrew J. Kueh, Craig D. Hyland, Tracy A. Willson, Liam Salleh, Carolyn A. de Graaf, Jeffrey J. Babon, Marco J. Herold, Nicos A. Nicola, Warren S. Alexander

**Affiliations:** 1 Blood Cells and Blood Cancer Division, The Walter and Eliza Hall Institute of Medical Research, Parkville, VIC 3052 Australia; 2 Department of Medical Biology, The University of Melbourne, Parkville, VIC 3010 Australia; 3 Present Address: Olivia Newton-John Cancer Research Institute, Heidelberg, VIC Australia; 4 Present Address: School of Cancer Medicine, La Trobe University, Heidelberg, VIC 3084 Australia

**Keywords:** Myeloproliferative disease, Cell signalling

## Abstract

Thrombopoietin (Tpo), which binds to its specific receptor, the Mpl protein, is the major cytokine regulator of megakaryopoiesis and circulating platelet number. Tpo binding to Mpl triggers activation of Janus kinase 2 (Jak2) and phosphorylation of the receptor, as well as activation of several intracellular signalling cascades that mediate cellular responses. Three tyrosine (Y) residues in the C-terminal region of the Mpl intracellular domain have been implicated as sites of phosphorylation required for regulation of major Tpo-stimulated signalling pathways: Mpl-Y565, Mpl-Y599 and Mpl-Y604. Here, we have introduced mutations in the mouse germline and report a consistent physiological requirement for Mpl-Y599, mutation of which resulted in thrombocytopenia, deficient megakaryopoiesis, low hematopoietic stem cell (HSC) number and function, and attenuated responses to myelosuppression. We further show that in models of myeloproliferative neoplasms (MPN), where Mpl is required for pathogenesis, thrombocytosis was dependent on intact Mpl-Y599. In contrast, Mpl-Y565 was required for negative regulation of Tpo responses; mutation of this residue resulted in excess megakaryopoiesis at steady-state and in response to myelosuppression, and exacerbated thrombocytosis associated with MPN.

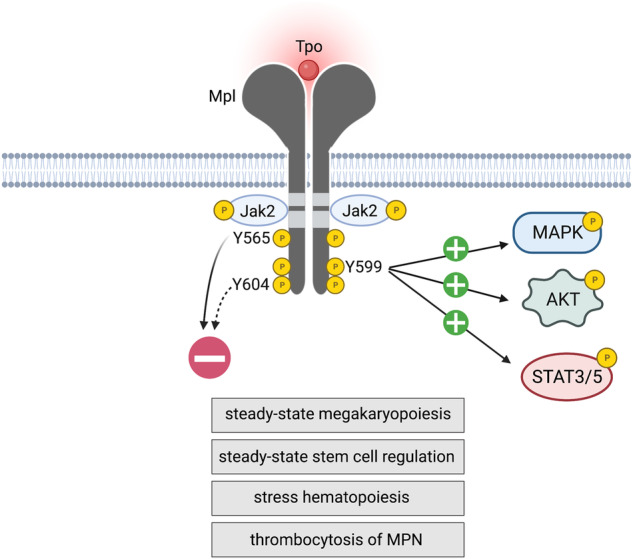

## Introduction

The maintenance of healthy numbers of blood platelets is dependent on the production of megakaryocytes, the specialised cells from which platelets are released. Megakaryocytes are produced from hematopoietic stem cells (HSC), either via transiting through intermediate progenitor cells, or under some circumstances deriving more directly from the HSC population [1]. Thrombopoietin (Tpo) is the major cytokine regulator of megakaryopoiesis, acting via Mpl, its specific cell surface receptor. Mice lacking Tpo or Mpl produce around 10% the normal numbers of megakaryocytes and platelets at steady-state [2, 3] and recovery of platelets in response to stresses such as bone marrow (BM) transplantation or cytotoxic drugs are attenuated [4–6]. Mpl signalling also plays a major role in maintaining normal HSC numbers and activity [4, 7, 8].

Binding of Tpo to Mpl initiates receptor signalling, initially via activation of Jak2, and phosphorylation of intracellular tyrosine residues, primarily the three most C-terminal, Y565, Y599 and Y604 (intracellular Y78, Y112 and Y117), that are implicated as docking/recruitment sites for signal transducers. Major signalling cascades activated by Tpo/Mpl include the Jak/STAT, PI3K-Akt and Ras-MAPK1/3 pathways [9–11]. Structure-function studies in vitro have identified intracellular residues and motifs implicated in signal transduction and Mpl regulation. Two motifs in the membrane proximal half of the Mpl intracellular domain, Box1 and Box2, are required for Jak2 binding and/or activation [10, 12]. Mpl-Y599 is associated with positive mediation of signalling, via recruitment and/or activation of Shc and Gab1/2, promoting Ras-MAPK and PI3K-Akt signalling respectively [13–16], but appears also to contribute to negative regulation via activation of Lyn kinase [17], which itself activates the E3 ubiquitin ligase c-Cbl [18]. The Lnk adaptor binds Mpl/Jak2, is phosphorylated by Jak2, and recruits c-Cbl, which targets Mpl and Jak2 for ubiquitination [18–20]. Lnk also recruits the BRISC de-ubiquitination complex, which controls association of Jak2 with cell-surface Mpl and thus Jak2 stability and activity [21]. Negative regulation of signalling has also been linked to Mpl-Y565, which is required for receptor internalisation, via interaction with the clathrin-recruiting adaptor AP2 [22], and via Syk binding, linked to suppression of the Ras-MAPK pathway [23]. Mpl-Y604 has been implicated in binding to Tensin2, a postulated adaptor for recruitment of PI3K [24]. Activation of STAT5, and also STAT3, is characteristic of Mpl signalling. Full activation of STATs has been linked to Mpl-Y599 and Mpl-Y604, although at least for STAT5, phosphorylation appears sometimes to occur in the absence of these residues [14, 16]. Consistent with a negative regulatory role for Mpl-Y565 and a positive role for Mpl-Y599, in mice reconstituted with bone marrow cells ectopically expressing a ligand-independent receptor (Mpl-W515A), Mpl-Y599F mutation compromised receptor activity, while Mpl-Y565F enhanced it [25].

Activation of the JAK/STAT pathway is integral to the development of the Philadelphia-negative myeloproliferative neoplasms (MPN), including polycythaemia vera (PV), essential thrombocythaemia (ET) and myelofibrosis (MF). Mutations in *JAK2* account for almost all PV and over 50% of ET and primary MF, with mutations in *CALR*, which result in activation of MPL, or mutations in *MPL* itself, accounting for much of the remainder [26]. Indeed, to drive MPN development, mutant forms of Jak2 and Calr require a functional homodimeric type I cytokine receptor such as Mpl [27–33].

Here, we have generated *Mpl* mutations in the mouse germline to study the roles of receptor intracellular domains and tyrosine residues in normal and pathophysiological actions of Tpo/Mpl signalling under endogenous regulation of receptor expression. We find a consistent physiological requirement for Mpl-Y599, mutation of which resulted in thrombocytopenia, deficient megakaryopoiesis, reduced HSC number and function, and attenuated responses to 5-fluorouracil (5-FU) treatment. Moreover, the thrombocytosis driven by MPN-associated mutations in *Jak2* and h*Calr* was dependent on intact Mpl-Y599. In contrast, Mpl-Y565 was required for attenuation of TPO responses; mutation resulted in excess megakaryopoiesis at steady-state and in response to 5-FU, and exacerbated mutant *Jak2* and h*Calr*-driven MPN.

## Results

### The C-terminal 85 amino acids of the Mpl receptor are required in vivo

**Fig. 1:**
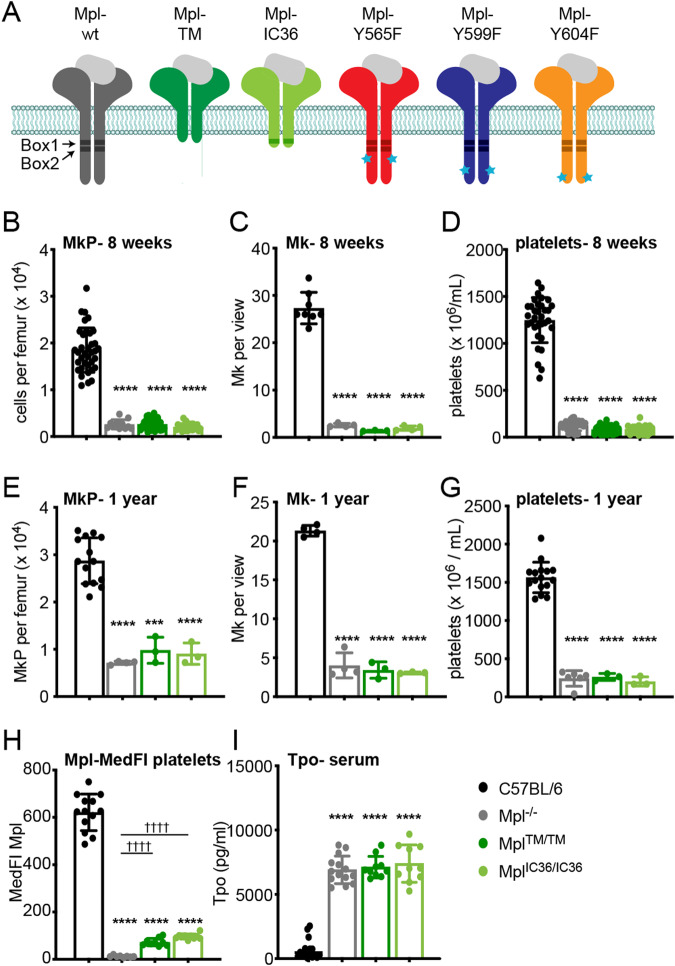
The Mpl C-terminal domain is required for megakaryopoiesis. **A** Wild-type and mutant Mpl receptors used in this study. Cytoplasmic Box1 and Box2 motifs are indicated by the shaded boxes and blue stars indicate the position of tyrosine residues mutated to phenylalanine. **B**–**G** Analysis of wild-type C57BL/6, *Mpl*^*−/−*^, *Mpl*^*TM/TM*^ and *Mpl*^*IC36/IC36*^ mice at 8-weeks (panels **B**–**D**) and 1-year of age (panels **E**–**G**). **B**, **E** Number of megakaryocyte progenitor cells (MkP, Lin^−^Sca1^−^Kit^+^CD150^+^CD41^+^) per femur. For panel **B**, *n* = 11–36 mice, for panel **E**, *n* = 3–14. **C**, **F** Number of megakaryocytes per scanned image enumerated from 10 non-overlapping images of histological sections of sternal BM per mouse (*n* = 3–8). **D**, **G** Platelet numbers. For panel **D**, *n* = 22–33, for panel G, *n* = 3–16. **H** Median fluorescence intensity (MedFI) of anti-Mpl antibody staining on platelets (*n* = 8–13). **I** Serum Tpo levels (*n* = 10–17). Each point represents data from an individual mouse, bars represent mean ± SD. ***P < 0.001; **** *P* < 0.0001 for comparison with C57BL/6; ^††††^ *P* < 0.0001 for comparison with *Mpl*^*−/−*^ (one-way ANOVA with Dunnett’s correction for multiple comparisons).

Initially, two mutants carrying deletions within the 121-amino acid Mpl cytoplasmic domain were investigated (Fig. 1A). Mpl-TM lacks all but one intracellular amino acid. Mpl-IC36 lacks the C-terminal 85 residues but retains the membrane-proximal 36 amino acids including the Box1 motif. Mice with mutations in the endogenous *Mpl* locus incorporating Mpl-IC36 or Mpl-TM were generated (*Mpl*^*TM/TM*^ and *Mpl*^*IC36/IC36*^). In both *Mpl*^*TM/TM*^ and *Mpl*^*IC36/IC36*^ mice, relative to wild type, significant reductions in the numbers of megakaryocyte progenitor cells (MkP), megakaryocytes (Mk) and platelets were observed, comparable to those in mice lacking the receptor (*Mpl*^*−/−*^) and evident at both 8 weeks and 1 year of age (Fig. 1B–G). At the surface of platelets and MkP, expression of Mpl-TM and Mpl-IC36 receptors was significantly reduced compared to wild-type (Fig. 1H, Supplementary Fig. 1A). Elevated serum Tpo was evident in *Mpl*^*TM/TM*^ and *Mpl*^*IC36/IC36*^ mice (Fig. 1I) consistent with low receptor expression and thus reduced receptor-mediated removal from the circulation [34]. This data establishes that in vivo, Box1 alone is insufficient for significant Mpl cell surface expression.

### Control of megakaryopoiesis in vivo by Mpl C-terminal tyrosine residues

**Fig. 2:**
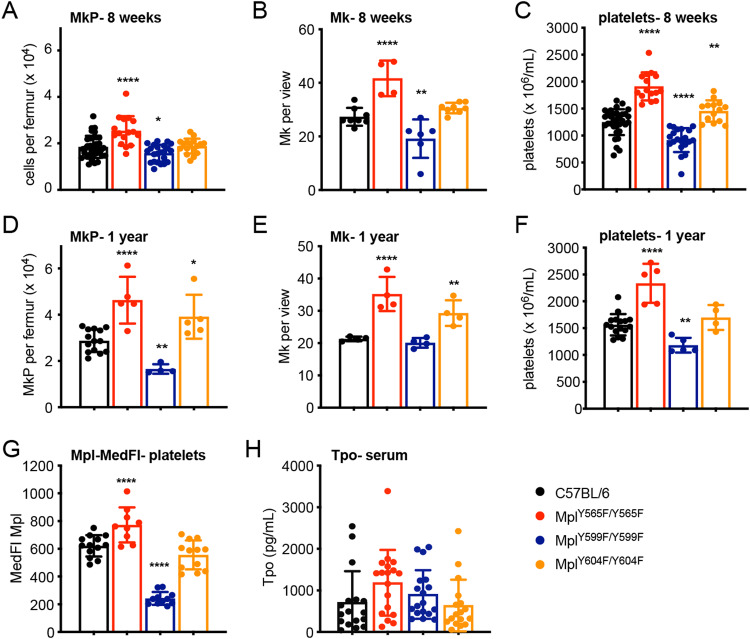
Role of Mpl tyrosine residues in megakaryopoiesis. Analysis of wild-type C57BL/6, *Mpl*^*Y565F/Y565F*^*, Mpl*^*Y599F/Y599F*^and *Mpl*^*Y604F/Y604F*^mice at 8-weeks (panels **A**–**C**) and 1-year of age (panels **D**–**F**). **A**, **D** Number of megakaryocyte progenitor cells (MkP, Lin^−^Sca1^−^Kit^+^CD150^+^CD41^+^) per femur. For panel **A**, *n* = 16–36, for panel **D**, *n* = 4–14. **B**, **E** Number of megakaryocytes per scanned image enumerated from 10 non-overlapping images of histological sections of sternal BM per mouse (*n* = 4–8). **C**, **F** Platelet numbers. For panel **C**, *n* = 14–33, for panel **F**, *n* = 4–16. **G** Median fluorescence intensity (MedFI) of anti-Mpl antibody staining on platelets (*n* = 9–13). **H** Serum Tpo levels (*n* = 17–18). Each point represents data from an individual mouse (in panels **A**–**F**, data from C57BL/6 mice is reproduced from Fig. 1 for comparison) and bars represent mean ± SD. *P < 0.05; **P < 0.005; ****P < 0.0001 for comparison with C57BL/6 (one-way ANOVA with Dunnett’s correction for multiple comparisons).

Next, we investigated the megakaryopoietic function of the three Mpl C-terminal tyrosine residues by introduction of individual tyrosine-to-phenylalanine mutations (Fig. 1A) into the mouse germline. In *Mpl*^*Y565F/Y565F*^ mice, increased numbers of MkP, Mk and platelets were evident at 8-weeks (Fig. 2A–C) and at one year of age (Fig. 2D–F). Expression of Mpl-Y565F on platelets, but not MkP was slightly increased compared to wild-type Mpl in C57BL/6 mice, but serum Tpo concentration remained unaltered (Fig. 2G, H, Supplementary Fig. 1A). In contrast, *Mpl*^*Y599F/Y599F*^ mice were thrombocytopenic at both 8-weeks and one year of age, variably accompanied by reduced Mk or MkP (Fig. 2A–F). Mpl-Y599F expression on platelets and MkP was reduced to a similar level as observed in Mpl heterozygous (*Mpl*^*+/-*^) mice, which of note show no reduction in platelet counts (Fig. 2G, Supplementary Fig. 1A–D), but serum Tpo levels in *Mpl*^*Y599F/Y599F*^ mice resembled C57BL/6 controls (Fig. 2H). The Mpl-Y604F mutation did not impact receptor expression or serum Tpo concentration (Fig. 2G, H, Supplementary Fig. 1A). A small increase in platelet number was observed in *Mpl*^*Y604F/Y604F*^ mice at 8 weeks, but not at 1 year of age, variably accompanied by modest increases in Mk or MkP numbers (Fig. 2A–F).

### Mpl tyrosine residues regulate hematopoietic stem cells

**Fig. 3:**
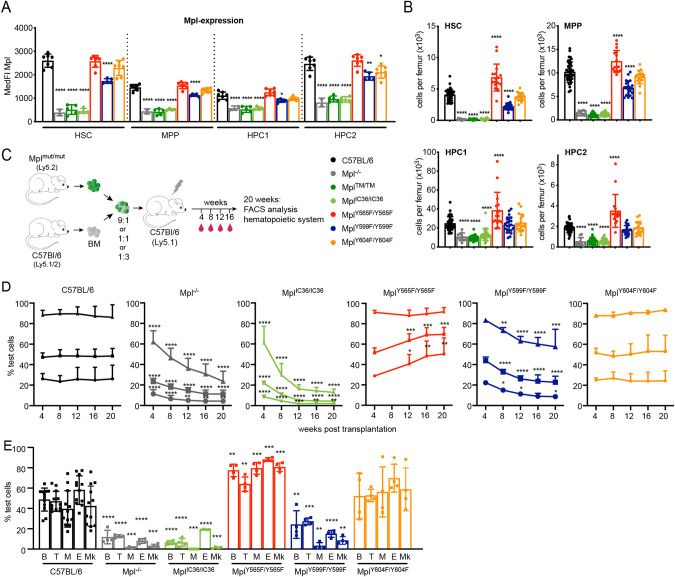
Hematopoietic stem cell number and function in *Mpl*-mutant mice. **A** Median fluorescence intensity (MedFI) of anti-Mpl antibody staining on HSC (Lin^−^Sca1^+^Kit^+^CD150^+^CD48^−^), MPP (Lin^−^Sca1^+^Kit^+^CD150^−^CD48^lo/−^), HPC1(Lin^−^Sca1^+^Kit^+^CD150^−^CD48^+^) and HPC2 (Lin^−^Sca1^+^Kit^+^CD150^+^CD48^+^) from *n* = 2–7 mice per genotype. **B** Total number of HSC, MPP1, HPC1 and HPC2 per femur in C57BL/6 and *Mpl*-mutant mice (*n* = 11–38). **C** Competitive transplantation design. Irradiated Ly5.1 recipient mice were transplanted with 9:1, 1:1 or 1:3 mixtures of test *Mpl*-mutant (Ly5.2):competitor wild-type (C57BL/6, Ly5.1/2) cells. Blood was analysed 4, 8, 12, 16 and 20 weeks after transplantation; reconstitution of other organs after 20 weeks. **D** Test contribution (% Ly5.2) to peripheral blood at the indicated timepoints after transplantation (*n* = 3–12 recipient mice per donor cell mixture, per timepoint; triangles: 9:1 test:competitor ratio; squares, 1:1 and circles, 1:3). **E** Test contribution (% Ly5.2) to B-cells (B, B220^+^); T-cells (T, CD4^+^ and/or CD8^+^); myeloid cells (M, CD11b^+^ and/or Gr1^+^ and/or Ly6G^+^ and/or F4/80^+^); erythroid cells (**E**, Ter119^+^); and megakaryocytes (Mk, CD41^+^) in mice transplanted at 1:1 test:competitor ratio (*n* = 4–12 per donor cell mixture, at 20 weeks). Each point is data from an individual mouse, bars represent mean ± SD. *P < 0.05; **P < 0.005; ***P < 0.001; ****P < 0.0001 for comparison with C57BL/6 (panels **A** and **B**) or C57BL/6 test-transplanted recipients at the same timepoint (panels **D** and **E**) using one-way ANOVA with Dunnett’s correction for multiple comparisons.

Because Mpl signalling plays a key role in HSC maintenance and activity we next analysed the expression of the Mpl-mutant receptors within the Lin^-^Sca1^+^Kit^+^ BM compartment. The SLAM markers CD150 and CD48 were used to define long-term self-renewing HSC, multipotential progenitor cells with limited self-renewal capacity (MPP) and non-renewing hematopoietic progenitor cells (HPC-1 and HPC-2) (Supplementary Table 1) [35]. In C57BL/6 mice, Mpl was expressed prominently on HSC and HPC-2, with lower levels on MPP and HPC-1 (Fig. 3A). Truncated Mpl receptors in *Mpl*^*TM/TM*^ and *Mpl*^*IC36/IC36*^ mice were expressed at negligible levels on these populations (Fig. 3A). For each of these cell populations, expression of the receptor in *Mpl*^*Y565F/Y565F*^ and *Mpl*^*Y604F/Y604F*^ mice was similar to wild-type, while expression was reduced in *Mpl*^*Y599F/Y599F*^ cells (Fig. 3A). RNAseq analysis on freshly-isolated LSK cells from Mpl-mutant mice revealed no transcriptional changes between wild-type and each of *Mpl*^*Y565F/Y565F*^, *Mpl*^*Y599F/Y599F*^ and *Mpl*^*Y604F/Y604F*^ cells under these unmanipulated conditions. Of note, this included levels of *Mpl* RNA (Supplementary Fig. 2A), suggesting that low cell surface expression of Mpl-Y599F is likely to be post-transcriptional.

*Mpl*^*Y565F/Y565F*^ mice produced significantly more HSC, MPP, HPC-1 and HPC-2 (Fig. 3B). In contrast, in *Mpl*^*Y599F/Y599F*^ mice, the numbers of HSC and MPP were reduced, while HPC-1 and HPC-2 were within the normal range (Fig. 3B). *Mpl*^*Y604F/Y604F*^ mice exhibited normal numbers of these populations (Fig. 3B). As observed in platelets and MkP (Supplementary Fig. 1C, D), receptor expression on *Mpl*^*Y599F/Y599F*^ stem cell populations was similar to that on *Mpl*^*+/*−^ cells, but the numbers of HSC were significantly reduced in *Mpl*^*Y599F/Y599F*^ mice compared with *Mpl*^*+/−*^ (Supplementary Fig. 1E, F). Thus, the key phenotypic deficiencies in *Mpl*^*Y599F/Y599F*^ mice – numbers of platelets and HSC, were evident relative to both wild-type and *Mpl*^*+/−*^ mice, despite similar levels of receptor expression on *Mpl*^*Y599F/Y599F*^ and *Mpl*^*+/−*^ cells.

Few changes were found in the numbers of common myeloid (CMP) and granulocyte-macrophage (GMP) progenitors, pre colony-forming units erythroid (preCFU-E) and CFU-E, as well as in numbers of B-cells, T-cells, neutrophils, erythroid cells and NK cells in the BM of mutant mice, with the exception of *Mpl*^*TM/TM*^ and *Mpl*^*IC36/IC36*^ mice, in which significant deficits were uniformly evident in progenitor cell populations (Supplementary Fig. 2B, C). Other than platelets, the numbers of other blood cells in the circulation were normal in each of the *Mpl*-mutant mice, with the exception of reduced red blood cell numbers in *Mpl*^*TM/TM*^ and *Mpl*^*IC36/IC36*^ mice (Supplementary Table 2). Flow cytometric assessment of hematopoietic progenitor cells was complemented with clonogenic assays. When using a broad myeloid stimulus of SCF + IL-3 + EPO the total numbers of colony forming cells (CFC) in the bone marrow, as well as the numbers of CFC with megakaryocytic potential, were similar to wild-type in *Mpl*-mutant mice, with the exception of reduced total CFC numbers in *Mpl*^*TM/TM*^ and *Mpl*^*IC36/IC36*^ mice (Supplementary Fig. 2D). In line with the reduction in MkP and Mk observered in *Mpl*^*Y599F/Y995F*^ mice the number of CFC with megakaryocytic potential were significantly reduced in these mice, when stimulated with IL3+Tpo, but not in *Mpl*^*Y565FY/565F*^*, Mpl*^*Y604F/Y604F*^ mice (Supplementary Fig. 2E).

HSC function was assessed using competitive reconstitution assays (Fig. 3C). Following transplantation of test BM cells mixed in various ratios with wild-type competitor cells, wild-type C57BL/6 test cells maintained a stable contribution to blood cell production over the 20-week assay period, which was in proportion to the relative amounts of test marrow injected (Fig. 3D, Supplementary Fig. 3A). In contrast, the contribution of *Mpl*^*IC36/IC36*^ cells to the blood was rapidly outcompeted even at 9-fold excess in the initial graft, as was also observed with *Mpl*^*−/−*^ cells. *Mpl*^*Y565F/Y565F*^ contribution to blood cell production increased over time, most evident in 1:1 and 1:3 test:competitor ratios. The percentage of *Mpl*^*Y599F/Y599F*^-derived blood cells declined steadily over time at all transplanted ratios, while *Mpl*^*Y604F/Y604F*^ contribution remained stable and similar to wild-type (Fig. 3D, Supplementary Fig. 3A). At 20 weeks after transplantation, *Mpl*^*Y565F/Y565F*^ donor marrow consistently out-competed wild-type cells in each of Mk, B-lymphoid, T-lymphoid, myeloid and erythroid cell populations; *Mpl*^*Y599F/Y599F*^ was uniformly less competitive and *Mpl*^*Y604F/Y604F*^ contribution was similar to that of wild-type cells (Fig. 3E, Supplementary Fig. 3B). As expected from the levels of blood cell chimerism, the contribution from *Mpl*^*IC36/IC36*^ and *Mpl*^*−/−*^ transplants to these populations was significantly less than wild-type cells after 20 weeks (Fig. 3E, Supplementary Fig. 3B). Myeloid reconstitution appeared particularly affected relative to that of lymphoid cells in recipients of *Mpl*^*Y599F/Y599F*^, *Mpl*^*IC36/IC36*^ and *Mpl*^*−/−*^ cells, suggesting a potential differentiation bias in Mpl-deficient cells, which may be consistent with previous studies that observed increased numbers of lymphoid cell populations at steady state in *Mpl*^*−/−*^ mice [36]. Together, these new in vivo insights demonstrate that, of the C-terminal Mpl Y-residues, only Mpl-Y599 is essential for replete megakaryopoiesis and HSC number and function. Mpl-Y565 has a significant negative regulatory role, while Mpl-Y604 contributes more moderately, and in contrast to previous over-expression studies in vitro, has a modest negative regulatory role in vivo.

### Mpl-Y599 is required for activation of signalling in Tpo-stimulated cells

**Fig. 4:**
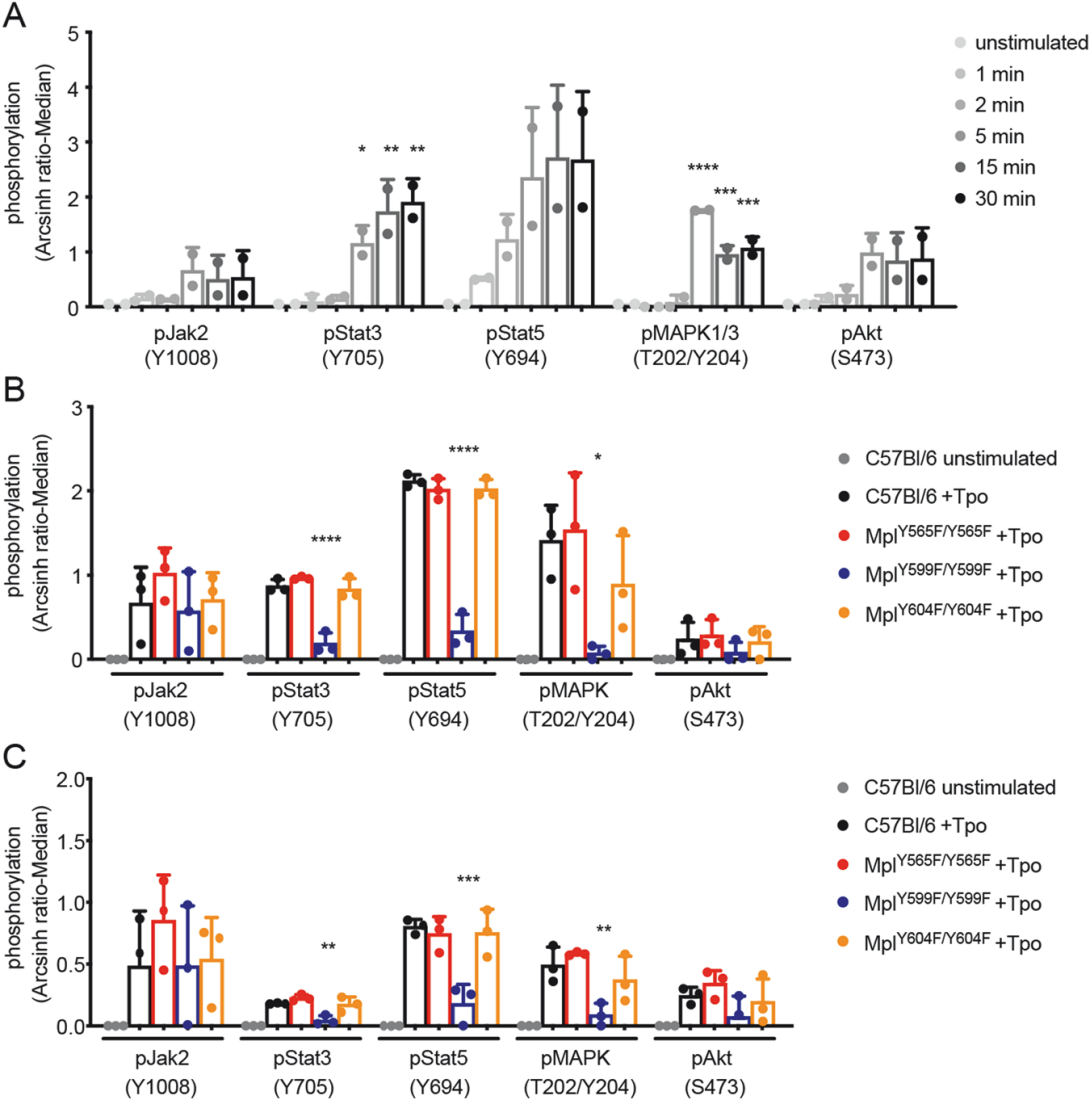
Signalling pathways activated in Tpo-stimulated *Mpl*-mutant cells. Detection of phospho(p)Jak2, pStat3, pStat5, pMAPK1/3 and pAkt by mass cytometry (expressed as arcsinh-transformed median intensity relative to unstimulated C57BL/6 cells) in **A** C57BL/6 HSC (Lin^−^Sca1^+^Kit^+^CD150^+^CD48^−^, *n* = 2) stimulated with 100 ng/ml Tpo for 1, 2, 5, 15 or 30 min and **B** C57BL/6 and *Mpl*-mutant HSC (*n* = 3) or **C** MkP (*n* = 3) stimulated with 100 ng/ml Tpo for 5 min. *P < 0.05, **P < 0.005, ***P < 0.001, ****P < 0.0001 for comparison of Tpo-stimulated cells at each timepoint with unstimulated C57BL/6 cells (panel **A**) or Tpo-stimulated *Mpl*-mutant cells with C57BL/6+Tpo (panels **B** and **C**) by one-way ANOVA with Dunnett’s correction for multiple comparisons.

Mass cytometry was used to examine activation of signalling pathways in Tpo-stimulated HSC. A pilot study in C57BL/6 HSC showed induction of phospho(p)Jak2, pSTAT3, pSTAT5, pMAPK1/3 and pAkt over a 30-min period following Tpo exposure in 2 independent experiments (Fig. 4A). A 5-min timepoint was chosen to examine signalling in Mpl-mutant cells. While responses in *Mpl*^*Y565F/Y565F*^ and *Mpl*^*Y604F/Y604*^ HSC were no different to C57BL/6 cells, in *Mpl*^*Y599F/Y599F*^ HSC significantly attenuated induction of pSTAT3, pSTAT5 and pMAPK1/3 was observed in response to Tpo stimulation (Fig. 4B, Supplementary Fig. 4A). Under these conditions, induction of pJak2 and pAkt by Tpo did not reach statistical significance in control or mutant cells. Similar observations were made in MkP stimulated with TPO (Fig. 4C). In view of the gain-of-function phenotype observed in *Mpl*^*Y565F/Y565F*^ mice, an extended timecourse of TPO stimulation and a TPO dose titration was performed: this also revealed no differences in the amounts of phosphorylated proteins in *Mpl*^*Y565F/Y565F*^ HSC relative to controls (Supplementary Fig. 4B, C). Thus, consistent with the requirement of Mpl-Y599 for normal HSC function and megakaryopoiesis, Mpl-Y599 is essential for activation of major TPO-stimulated signalling pathways. Interestingly, no apparent change in these pathways accompanied enhanced megakarypopoiesis in *Mpl*^*Y565F/Y565F*^ mice and, in contrast to in vitro studies linking Mpl-Y604 to activation of STAT signalling, these pathways were activated normally in *Mpl*^*Y604F/Y604F*^ HSC.

### Control of emergency hematopoiesis by Mpl C-terminal tyrosine residues

**Fig. 5:**
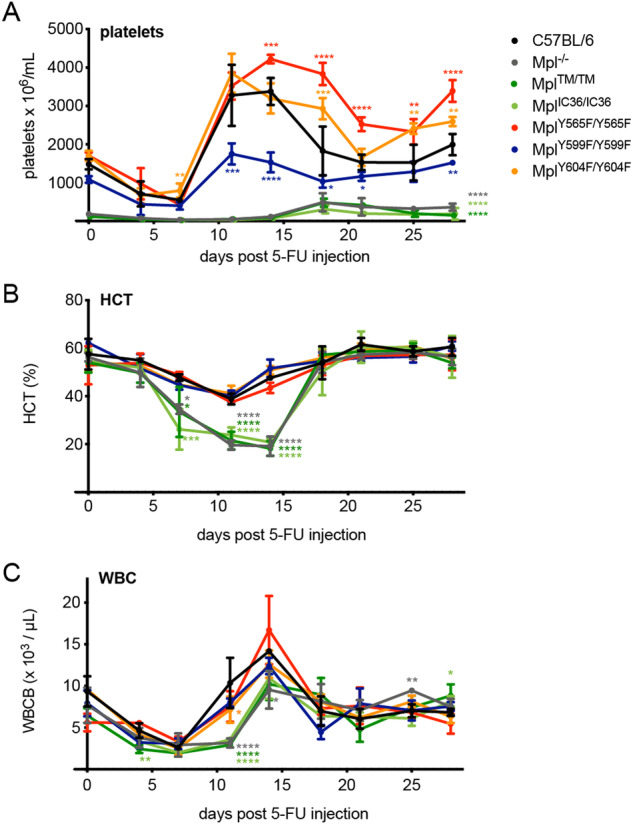
Responses to 5-FU-induced myelosuppression in *Mpl*-mutant mice. **A** Platelet numbers, **B** haematocrit (HCT), and **C** white blood cell numbers (WBC) in wild-type C57BL/6 and *Mpl*-mutant mice at indicated time points after 5-FU treatment. Data is presented as mean ± SD (*n* = 3–6 mice per genotype per timepoint). *P < 0.05; **P < 0.005; ***P < 0.001, ****P < 0.0001 for comparison with C57BL/6 at each time point by one-way ANOVA with Dunnett’s correction for multiple comparisons. Platelet counts for *Mpl*^*−/−*^, *Mpl*^*TM/TM*^ and *Mpl*^*IC36/IC36*^ mice were significantly reduced at all analysed timepoints (day 4, *P* < 0.05; day 7–28, *P* < 0.0001).

5-FU administration in C57BL/6 mice caused a rapid thrombocytopenia, followed by a transient rebound thrombocytosis with platelet counts ~2–3-fold higher than baseline (Fig. 5A). In *Mpl*^*Y565F/Y565F*^ mice, peak platelet numbers following 5-FU were significantly higher than in control mice and rebound thrombocytosis persisted. In contrast, in *Mpl*^*Y599F/Y599F*^ mice, the rebound response to 5-FU was significantly attenuated: peak platelet counts were no higher than 1.5-fold that of pre-treatment. In *Mpl*^*Y604F/Y604F*^ mice, the peak platelet number was no different to that in control mice; however, elevated platelet counts persisted during rebound (Fig. 5A). In *Mpl*^*TM/TM*^ or *Mpl*^*IC36/IC36*^ mice, an extended exacerbation of the steady-state thrombocytopenia was evident with a delayed recovery of platelet numbers, a response similar to that in *Mpl*^*−/−*^ mice (Fig. 5A). The effects of 5-FU on leukocytes and red blood cells were similar in each of the Mpl-tyrosine mutant mice to C57BL/6 controls; in *Mpl*^*TM/TM*^, *Mpl*^*IC36/IC36*^ and *Mpl*^*−/−*^ mice an extended and more severe anaemia was evident prior to recovery, as was an extended period of leukopenia (Fig. 5B, C).

### MPN development is dependent on Mpl-Tyr599 and attenuated by Mpl-Tyr565

**Fig. 6:**
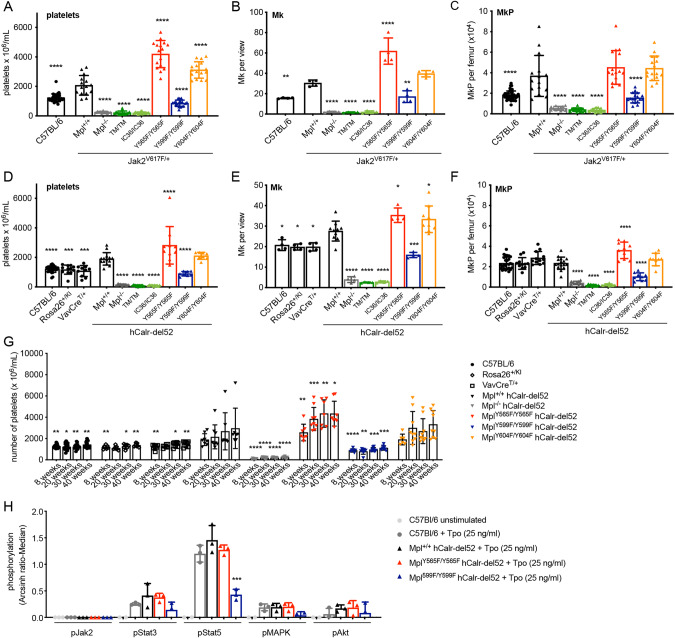
Effect on Jak2-V617F- and hCalr-del52-induced myeloproliferation in Mpl-mutant mice. **A** Platelet numbers (*n* = 12–37 per genotype), **B** number of megakaryocytes per scanned image enumerated from 10 non-overlapping images of histological sections of sternal BM per mouse (*n* = 4) and **C** number of megakaryocyte progenitor cells (MkP, Lin^−^Sca1^−^Kit^+^CD150^+^CD41^+^) per femur in C57BL/6, *Jak2*^*V617F*^ (*Mpl*^*+/+*^*;Jak2*^*V617F/+*^) and compound *Mpl*-mutant;*Jak2*^*V617F*^ mice at 8 weeks of age (*n* = 12–45). **D** Platelet numbers (*n* = 8–28), **E** number of megakaryocytes per scanned image enumerated from 10 non-overlapping images of histological sections of sternal BM per mouse (*n* = 4–10) and **F** number of MkP per femur in *hCalr-del52* (*Mpl*^*+/+*^;*hCalr-del52*) and compound *Mpl-*mutant;*hCalr-del52* mice and in C57BL/6, *Rosa26*^*+/KI*^ and *VavCre*^*T/+*^ control mice at 8 weeks of age (*n* = 8–26). **G** Platelet number over time in a cohort of *hCalr-del52*, compound *Mpl*-mutant;*hCalr-del52* and control mice (wild-type C57BL/6, Rosa26^+/KI^, VavCre^T/+^) (*n* = 6–18). **H** Detection of pJak2, pStat3, pStat5, pMAPK1/3 and pAkt by mass cytometry (expressed as arcsinh-transformed median intensity relative to unstimulated C57BL/6 cells) in *hCalr-del52* (*Mpl*^*+/+*^;*hCalr-del52*), compound *Mpl-*mutant;*hCalr-del52* and C57BL/6 control HSC (Lin^−^Sca1^+^Kit^+^CD150^+^CD48^−^, *n* = 2–3) stimulated with 25 ng/ml Tpo for 5 min. Each point represents data from an individual mouse; bars represent mean ± SD. *P < 0.05, **P < 0.005, ***P < 0.001, ****P < 0.0001 for comparison with *Mpl*^*+/+*^*;Jak2*^*V617F/+*^ or *Mpl*^*+/+*^;*hCalr-del52* at each time point (**A**–**G**) or for comparison of Tpo-stimulated C57Bl/6 cells (**H**) by one-way ANOVA with Dunnett’s correction for multiple comparisons.

To define the roles of Mpl signalling domains in MPN development, we generated mouse models of *Jak2*- and *Calr*-mutant disease. *Jak2*^*V617F/+*^ mice developed excess platelet numbers accompanied by significantly increased numbers of Mk and MkP, as well as an excess of red and white blood cells (Fig. 6A–C, Supplementary Fig. 5A, B, Supplementary Table 2). As shown previously [28], upon complete deletion of *Mpl*, Jak2-V617F expression did not increase platelet numbers: *Mpl*^*−/−*^*;Jak2*^*V617F/+*^ mice displayed thrombocytopenia and low numbers of Mk and MkP typical of *Mpl*^*−/−*^ mice (Fig. 6A–C, Supplementary Fig. 5A). *Mpl*^*TM/TM*^*;Jak2*^*V617F/+*^ and *Mpl*^*IC36/IC36*^*;Jak2*^*V617F/+*^ mice phenocopied *Mpl*^*−/−*^*;Jak2*^*V617F/+*^ mice (Fig. 6A–C). In contrast, *Mpl*^*Y565F/Y565F*^*;Jak2*^*V617F/+*^ mice developed an exacerbated phenotype, with platelet counts and numbers of megakaryocytes ~2-fold higher than in *Mpl*^*+/+*^*;Jak2*^*V617F/+*^ mice (Fig. 6A, B, Supplementary Fig. 6A). Platelet numbers in *Mpl*^*Y604F/Y604F*^*;Jak2*^*V617F/+*^ mice were also elevated relative to *Jak2*^*V617F/+*^, albeit to a lesser extent, although numbers of megakaryocytes and Mk progenitor cells were not increased (Fig. 6A–C). Strikingly, mutation of Mpl-Y599 prevented *Jak2*^*V617F*^-induced thrombocytosis, with platelet, megakaryocyte and MkP numbers in *Mpl*^*Y599F/Y599F*^*;Jak2*^*V617F/+*^ mice similar to that in parental *Mpl*^*Y599F/Y599F*^ animals (Fig. 6A–C, Supplementary Fig. 5A, Supplementary Fig. 6A).

In a second model of MPN, in which a *hCalr-del52* transgene was expressed in hematopoietic cells, significantly increased numbers of platelets, but not of other blood cells, were observed at 8 weeks of age, accompanied by increased numbers of Mk (Fig. 6D–F, Supplementary Fig. 5C, D, Supplementary Table 2). Consistent with a previous Calr-del52-driven mouse model [30], loss of the Mpl receptor in *Mpl*^*−/−*^;*hCalr-del52* mice prevented the excess megakaryopoiesis: mice developed the low numbers of platelets, megakaryocytes and MkP typical of *Mpl*^*−/−*^ mice and this was also the case in *Mpl*^*TM/TM*^*;hCalr-del52* and *Mpl*^*IC36/IC36*^*;hCalr-del52* mice (Fig. 6D–F, Supplementary Fig. 5C). Mutation of Mpl-Y565 exacerbated the *hCalr-del52* phenotype: *Mpl*^*Y565F/Y565F*^*;hCalr-del52* mice developed more platelets, megakaryocytes and MkP relative to *hCalr-del52* alone (Fig. 6D–F, Supplementary Fig. 5C). *Mpl*^*Y604F/Y604F*^*;hCalr-del52* mice showed increased numbers of megakaryocytes relative to *hCalr-del52* mice, but platelet and MkP numbers were not elevated (Fig. 6D–F, Supplementary Fig. 5C). Strikingly, mutation of Mpl-Y599 in *Mpl*^*Y599F/Y599F*^*;hCalr-del52* mice completely abrogated the *hCalr*^*del52*^ phenotype, with platelet, Mk and MkP counts no higher than in *Mpl*^*Y599F/Y599F*^ controls (Fig. 6D–F, Supplementary Fig. 5C). The phosphorylation of signal transducers in response to a reduced dose (25 ng/ml) of Tpo in HSC from *hCalr-del52* and *Mpl*^*Y565F/Y565F*^*;hCalr-del52* HSC was no different to that in wild type HSC, while induction of Stat5 phopshorylation was attenuated in *Mpl*^*Y599F/Y995F*^*;hCalr-del52* HSC (Fig. 6H).

With age, the thrombocytosis in *Mpl*^*Y565F/Y565F*^*;hCalr-del52* mice progressed (Fig. 6G) and examination of histological sections revealed that of 10 mice examined, 5 had developed a myelofibrosis-like pathology in the BM that was observed in only 1 of 8 *hCalr-del52* and 1 of 9 *Mpl*^*Y604F/Y604F;*^*hCalr-del52* mice (Supplementary Fig. 6B). There was no change in platelet number nor evidence of fibrosis in *Mpl*^*Y599F/Y599F*^*;hCalr-del52* mice at 40 weeks of age (*n* = 8, Fig. 6G, Supplementary Fig. 6B). Thus, these data confirm the previously described dependence of Jak2-V615F- and Calr-del52-driven thrombocytosis on an intact Mpl receptor and establish that Mpl-Y599 is the crucial signalling residue for disease. Furthermore, even in the context of deregulated Mpl activity driven by mutant Jak2 and Calr, the negative regulatory function of Mpl-Y565 is intact in MPN, albeit unable to fully normalise platelet numbers.

## Discussion

Studies in cell lines have been indispensable in defining Mpl signal transduction pathways and the roles of intracellular receptor domains and residues, but inevitably involve ectopic receptor expression, often at markedly higher levels than normal, and often in cells that are not related to the physiological context in which Tpo and Mpl act in vivo. Here, we generated germline mutations in the mouse *Mpl* gene allowing definitive insights into the roles of specific domains and intracellular tyrosine residues in vivo in the context of endogenous control of receptor expression, both in healthy hematopoiesis and MPN.

Sequences in the membrane proximal region of the intracellular Mpl domain, known as Box1 and Box2, are implicated in binding and activation of Jak2, the initial impetus for Tpo-activated Mpl signalling. Moreover, in cell lines, the Jak2-Mpl interaction is required for cell-surface receptor expression, with Jak2 stabilising the mature receptor form. Box1 is proposed to be the primary Jak2 interaction site with Box2 further required for efficient cell surface expression [37, 38]. In mice, we found that Mpl-IC36, a receptor retaining only 36 membrane-proximal amino acids including Box1 but not Box2, was expressed at very low or undetectable levels on HSC or platelets. Accordingly, *Mpl*^*IC36/IC36*^ mice were indistinguishable from *Mpl*^*−/−*^ mice: steady-state megakaryopoiesis was compromised, recovery of platelet numbers following 5-FU was attenuated, HSC function was defective, and *Mpl*^*IC36/IC36*^ mice failed to develop features of MPN driven by mutations in *Jak2* or *Calr*. Previous analysis of knockin mice lacking the C-terminal 60 cytoplasmic Mpl residues (Δ60 mice), retaining both Box1 and Box2, reported normal receptor expression [6]. Together, these data suggest that regulation of Mpl in vivo requires the full domain encompassing Box1 and Box2: retention of only Box1 is insufficient for significant cell surface expression.

In MplΔ60 mice, numbers of megakaryocytes and platelets were normal at steady-state, although platelet recovery from myelosuppression was attenuated, as was HSC activity [6, 39]. In these previous studies, specific in vivo roles of the 3 C-terminal tyrosine residues could not be assessed. To address this, we generated mice with germline mutations in these tyrosine residues, each of which is reported to be phosphorylated upon TPO stimulation in various contexts [12, 14, 23]. A key finding from our study was that only mutation of Mpl-Y599 resulted in signalling defects and phenotypic deficiencies. While Jak2 was activated relatively normally in TPO-stimulated *Mpl*^*Y599F/Y599F*^ cells, activation of STAT3 and STAT5, as well as MAPK1/3 were all attenuated in HSC. Deficiencies in all TPO-dependent phenotypes were observed: *Mpl*^*Y599F/Y599F*^ mice were thrombocytopenic and produced fewer than normal HSC, and responses to myelosuppressive challenge were compromised, as was the capacity of *Mpl*^*Y599F/Y599F*^ BM to reconstitute transplant recipients. In contrast, mutation of Mpl-Y565 resulted in thrombocytosis, excess megakaryopoiesis and increased HSC production and activity, as well as augmented platelet recovery from 5-FU treatment, observations consistent with the identification of TPO-hypersensitive mutations at this tyrosine residue in human MPN [40, 41]. These amplified TPO-dependent phenotypes were not accompanied by an increase in activation of the Jak/STAT, MAPK and Akt pathways in primary *Mpl*^*Y565F/Y565F*^ HSC or MkP at times shortly after maximal TPO stimulation, nor by increased sensitivity of these signalling pathways to lower TPO concentrations. Thus, enhanced signalling, as observed in cell lines expressing Mpl-Y565F [16, 23] was not observed in primary *Mpl*^*Y565F/Y565F*^ cells, but over the extended periods that responses develop in vivo, subtle differences in signalling may translate into significant phenotypic effects or may involve other pathways. Mpl-Y565 is implicated in regulation of receptor internalisation, via interaction with the clathrin-recruiting adaptor AP2 [22], and Syk binding [23]. This role is consistent with the increased levels of Mpl-Y565F evident on *Mpl*^*Y565F/Y565F*^ platelets and likely contributes to augmented TPO-dependent phenotypes in these mice. Reduced expression of Mpl-Y599F on platelets and LSK subsets was noted, which may also contribute to deficiencies in *Mpl*^*Y599F/Y599F*^ mice, although expression levels were no lower than that of *Mpl*^*+/-*^ mice, in which platelet and HSC numbers are not reduced. Interestingly, despite changes in circulating platelet numbers, the concentration of circulating TPO in *Mpl*^*Y565F/Y565F*^ and *Mpl*^*Y599F/Y599F*^ mice was similar to that observed in mice with wild-type Mpl. TPO concentration is typically inversely related to platelet number due to receptor-mediated uptake [34], as clearly evident in *Mpl*^*TM/TM*^, *Mpl*^*IC36/IC36*^ and *Mpl*^*−/−*^ mice, each of which displays marked thrombocytopenia and a corresponding elevation in circulating TPO concentration. It is likely that the less severe magnitude of change in platelet numbers accounts for the absence of significant effects on TPO levels in *Mpl*^*Y565F/Y565F*^ and *Mpl*^*Y599F/Y599F*^ mice. Finally, mutation of Mpl-Y604 resulted in no discernable effect on activation of TPO-dependent signalling pathways; however in *Mpl*^*Y604F/Y604F*^ mice, some modest effects were noted such as mild thrombocytosis at steady state, a prolonged response to 5-FU and modest selective enhancement of platelet or megakaryocyte numbers in models of MPN. Thus, while Mpl-Y604 may contribute, its contributions in the biological contexts investigated appear moderate compared with Mpl-Y565 and Mpl-Y599 and, in contrast to in vitro analyses, Mpl-Y604 appears to confer a negative regulatory role in vivo.

Unlike trends observed in cell lines, we found that in vivo the major TPO-stimulated signalling pathways were not differentially associated with specific tyrosine residues; rather the Jak/STAT, MAPK and Akt pathways all depended upon Mpl-Y599 for wild-type levels of activation. Consistent with this, all the TPO-dependent biological responses measured were reduced in *Mpl*^*Y599F/Y599F*^ mice. It is not clear how potentially competing interactions might be reconciled for simultaneous activation of multiple signalling pathways recruited to a single phosphorylated tyrosine residue and further studies are warranted. Nevertheless, combined with the MplΔ60 phenotype [6, 39], these results are consistent with a model in which the membrane proximal half of the Mpl intracellular domain is sufficient for basal activation of the major signalling cascades, likely due in significant measure to its role in binding and activating Jak2, and for basal steady-state megakaryopoiesis, with the C-terminal Mpl domain responsible for fine-tuning the TPO response, particularly under acute need, with Mpl-Y599 augmenting basal activity and Mpl-Y565 coordinating negative regulation. Studies of the EPO receptor also show that intracellular tyrosine residues are dispensable for erythropoiesis at steady-state [42], suggesting potential similarities in modulatory rather than essential roles for tyrosine phosphorylation in both EPO-R and Mpl.

As described in previous mouse models [28, 30], we found that the excess megakaryopoiesis and thrombocytosis driven by expression of Jak2-V617F or Calr-del52 were ablated in the absence of Mpl. Strikingly, the ability of Jak2-V617F and Calr-del52 to drive pathogenic megakaryopoiesis was absolutely dependent on Mpl-Y599: numbers of platelets, megakaryocytes and MkP in *Mpl*^*Y599F/Y599F*^*;Jak2*^*V617F/+*^ and *Mpl*^*Y599F/Y599F*^*;hCalr*^*del52/+*^ mice were no higher than in *Mpl*^*Y599F/Y599F*^ mice themselves, and this was associated with reduced activation of Stat5 in *Mpl*^*Y599F/Y599F*^*;hCalr*^*del52/+*^ HSC. This is consistent with a previous observation of loss of Calr-del52-driven thrombocytosis on the MplΔ60 background [43]. Together, these data suggest that interaction between Jak2 and Mpl, dependent on the membrane-proximal intracellular receptor domain and sufficient for basal signalling and platelet production, is insufficient for Jak2-V617F or Calr-del52-driven thrombocytosis. Rather, they imply that efficient Mpl-Y599-mediated activation of major signalling pathways is required. This interpretation is consistent with the increased efficacy observed when Jak inhibitors are combined with inhibitors of MAPK or mTOR to reduce features of MPN in mouse models and/or patient cells [44–46]. Thrombocytosis was exacerbated in both MPN models on a *Mpl*^*Y565F/Y565F*^ background implying that the negative regulatory actions of Mpl-Y565 operate in diseased, as well as normal megakaryopoiesis. Previously published mouse models of JAK2-V617F MPN have variously displayed features of PV and/or ET, depending on whether mouse or human JAK2-V617F was used and the amount of mutant allele expression [47]. A previous report of *Mpl*^*−/−*^*;JAK2-V617F* mutant mice utilised a model without erythrocytosis [28]. In addition to thrombocytosis, our *Jak2*^*V617F/+*^ mice did develop erythrocytosis, which persisted in the absence of c-Mpl (Supplementary Table 2); this is likely to depend on activation of the EPO-R by mutant JAK2 as previously suggested [27, 31]. Finally, our data are consistent with the observation that in bone marrow reconstitution studies with cells ectopically expressing Mpl-W515A, an active version found in human MPN, Mpl-Y599F co-mutation prevented development of MPN, while Mpl-Y565F exacerbated disease [25], and extend these observations to otherwise wild-type receptors expressed at endogenously regulated levels in the context of Jak2 and hCalr mutant MPN.

Our analyses could not distinguish between the role of Mpl tyrosine residues in normal megakaryopoiesis and HSC regulation compared to its contribution to the abnormal hematopoiesis of MPN. All the Mpl-dependent phenotypes examined at steady-state, in response to stress, or the pathological megakaryopoiesis driven by Jak2-V617F or Calr-del52, were dependent on Mpl-Y599 and attenuated by Mpl-Y565. While effectively ameliorating symptoms, Jak inhibitors have not proven effective in eliminating disease [48]. The dependence of aspects of MPN on Mpl function has raised the prospect that Mpl may provide an effective alternative or complementary target for therapy [49]. We acknowledge that our studies have focussed on murine Mpl, and that differences with human Mpl may exist in the context of MPN, for example species differences in the degree of Mpl activation by mutant CALR [50] and that distinct conformations of human Mpl, but not the murine receptor, have been shown to mediate activation of wild-type JAK2 and JAK2-V617F [51]. Nevertheless, these studies do not themselves imply species differences in activation of specific signalling pathways downstream of JAK2 in normal versus MPN cells, which our data suggest are similar. In this case, new therapies for MPN may be rewarded by a focus on agents that specifically target the mutant forms of JAK2 or CALR, or that specifically disrupt their productive interaction with Mpl, rather than on targeting pathways common to both normal and diseased Mpl signalling where there may be a significant challenge of disrupting diseased cells while sparing normal hematopoiesis. Indeed, manipulating Mpl conformation to differentially modulate normal and mutant JAK2 activation may provide an effective therapeutic window in human disease and extracellular ligands that modulate human Mpl conformation have already been described [52].

## Methods

### Mice

Experimental procedures were approved by the Walter and Eliza Hall Institute of Medical Research Animal Ethics Committee. Mice were generated on a C57BL/6 background as previously described [53]. To generate *Jak2*^*V617F*^ and *Mpl*-mutant mice (Y565F, Y599F, Y604F, numbered as described [13, 54]), 1 or 2 guide-RNAs (gRNA) were used to create double stranded breaks within the target locus to stimulate homologous recombination and incorporation of a donor, mutation-carrying oligonucleotide. A solution containing 20 ng/μl of Cas9 mRNA, 10 ng/μl gRNA(s) and 40 ng/μl of the donor oligonucleotide (Supplementary Table 3) was injected in a continuous stream into the cytoplasm of fertilised one-cell embryos. Twenty-four hours later, two-cell embryos were transferred into pseudo-pregnant female mice. Offspring were genotyped by sequencing. To generate *Rosa26*^*hCalrdel52/+*^mice, a hCalr-del52-IRES-GFP cassette was incorporated into the *Rosa26* locus for Cre recombinase-dependent expression of hCalr-del52 cDNA, with GFP as a marker of expression, by insertion of the cDNA sequence via the AscI site into the CTV targeting vector (addgene #15912) (Supplementary Fig. 7A). A solution containing 20 ng/μl of Cas9 mRNA, 10 ng/μl gRNA (5’-CTCCAGTCTTTCTAGAAGAT-3’) and 5 ng/μl of the hCalr-del52-IRES-GFP plasmid was injected into embryos and mice generated as above. For all mice NGS amplicon sequencing was used to verify the insertion [55]. hCalr-del52 expression was targeted to hematopoietic cells with a VavCre transgene [56]. As expected, *Rosa26*^*hCalrdel52/+*^*;VavCre*^*T/+*^ mice (designated *hCalr-del52*) expressed GFP in hematopoietic cells. In a proportion of mice, distinct GFP^low^ and GFP^hi^ populations were evident in BM (8 of 13 mice) and platelets (13 of 16 mice), varying from negligible to over 80% GFP^hi^ (Supplementary Fig. 7B). The level of GFP expression correlated with expression of hCalr-del52 in western blots of BM lysates and with platelet count in individual mice (Supplementary Fig. 7C, D), consistent with the degree of thrombocytosis reflecting the level of hCalr-del52 expression in this model.

### Bone marrow transplants and 5-FU treatment

For competitive transplants, nucleated test BM cells (Ly5.2) were mixed with compound heterozygous Ly5.1/Ly5.2 competitor cells at 9:1, 1:1 or 1:3 test:competitor ratios. For each mixture, a total of 2 ×10^6^ cells was intravenously injected into irradiated (2 doses of 4.5 Gy, 3 h apart) Ly5.1 recipient mice. 5-FU (Hospira) was administered by intravenous injection at a dose of 150 mg/kg.

### Colony forming assays

For clonal analysis, a single cell suspension of bone marrow cells was prepared and 2.5 × 10^4^ cells were cultured in 1 mL semisolid agar cultures of 0.3% agar in Dulbecco’s modified Eagles medium, 20% newborn calf serum, containing either stem cell factor (100 ng/mL), erythropoietin (2 U/mL), and interleukin-3 (10 ng/mL) or interleukin-3 (10 ng/mL) and Tpo (500 ng/ml). Cultures were incubated at 37 °C for 7 days in a fully humidified atmosphere of 10% CO_2_ in air. Cultures were fixed, dried onto glass slides, and stained for acetylcholinesterase, Luxol fast blue, and haematoxylin, and the number and type of colonies were determined.

### Western blots

Cells were washed and lysed in 1% TritonX-100, 150 mM NaCl, 50 mM Tris HCl (pH7.4), 1 mM EDTA, 1 mM phenylmethylsulphonylfluoride (PMSF), 2 mM Na_3_VO_4_, 10 mM NaF and complete protease inhibitors (Roche). Proteins were separated in 4–12% Bis-Tris NuPAGE protein gels (Invitrogen) under reducing conditions, transferred to Immobilon-P membrane (Millipore) and immunoblotted with primary antibodies (Supplementary Table 4), followed by secondary HRP-conjugated antibody and visualised by enhanced chemiluminescence.

### Haematology and histological analysis

Blood was collected into EDTA coated tubes (Sarstedt) and analysed on an Advia 2120i haematological analyser (Siemens). Tpo in serum was measured by Quantikine ELISA (R&D Systems). Spleens and sternums were fixed in 10% neutral buffered formalin, sternums were decalcified, and the tissues were embedded in paraffin. Hematoxylin and eosin-stained sections were prepared and scanned on a bright field slide scanner (Pannoramic scan II, 3DHistech). For each tissue, megakaryocytes were counted from 10 non-overlapping images taken at a 20× magnification.

### Flow cytometry

Single-cell suspensions were prepared and for blood samples erythrocytes were lysed in 156 mM NH_4_Cl, 11.9 mM NaHCO_3_, 0.097 mM EDTA. Cells were stained with fluorophore-conjugated antibodies (Supplementary Table 4) on ice and analysed on a LSRFortessa or LSRII (Becton Dickinson). The cell surface markers used to define specific populations are provided in Supplementary Table 1. Representative profiles and gating strategies are shown in Supplementary Fig. 8. Cell sorting was performed on a FACSAria III. Dead cells were excluded by FluoroGold (AAT Bioquest) and data analysis performed using FlowJo10.4 (Becton Dickinson).

### Mass cytometry

Single-cell BM suspensions were enriched for stem and progenitor cells by negative selection of mature cells using EasySep Mouse Hematopoietic Progenitor Cell Isolation Kits (Stem Cell Technologies). Cells were stained with metal-tagged antibodies to cell surface markers (Supplementary Table 4), starved for 30 min at 37 ˚C and stimulated with the indicated concentration of Tpo. Cells were fixed in 1.6% paraformaldehyde for 10 min and permeabilised in methanol for 15 min on ice, stained with antibodies specific for phosphorylated proteins (Supplementary Table 4) and incubated overnight in 1.6% paraformaldehyde with Cell-IDTM Intercalator-Ir (Fluidigm). Samples were analysed on a Helios mass cytometer (Fluidigm). Data was analysed using Cytobank Premium (Beckman Coulter Life Sciences).

### RNA-seq

LSK cells sorted from 4–12 Mpl-mutant mice were pooled for each replicate and total RNA was extracted using the RNeasy Plus Micro kit (Qiagen). 50 ng RNA was used to generate cDNA libraries using Stranded mRNA kits (Illumina). Sequencing was performed on a NovaSeq sequencing system (Illumina) to generate 100 bp single-end reads. Reads were aligned to the GRCm38/mm10 build of the *Mus musculus* genome using Rsubread [57] aligner function and assigned to genes with featureCounts [58] before summarising as read counts per million (CPM) at gene level. Counts were transformed to log2-CPM and the mean-variance relationship estimated using the *voom* function in limma [59]. Library sizes were TMM normalised and differential expression was assessed using quasi-likelihood F-tests [60]. Genes were called differentially expressed if they achieved a false discovery rate of 0.05.

### Statistical analysis

Mice were selected based on genotype and otherwise randomly distributed into experimental groups. Both male and female mice were used. Investigators were not routinely blinded to experimental groups. Statistical significance was analysed using one-way ANOVA with correction for multiple comparison of three or more groups, two-sided t-tests for pairwise comparisons, and Pearson correlation coefficient as indicated in the Figure legends (GraphPad Prism Software Version 8). *P < 0.05; **P < 0.005; ***P < 0.001, ****P < 0.0001. Data are presented as mean ± SD.

### Supplementary information

Supplemental material

## Data Availability

RNA-seq data has been deposited under the Accession Number GSE233693.
