# Supplementary material for: Differential in vivo roles of Mpl cytoplasmic tyrosine residues in murine hematopoiesis and myeloproliferative disease
Source: Leukemia. 2024 Mar 15;38(6):1342–52. doi: 10.1038/s41375-024-02219-5 (PMC11147766; doi:10.1038/s41375-024-02219-5)

**Supplementary Data**

**Supplementary Tables**

**Supplementary Table 1. Cell surface markers used to define hematopoietic cell populations**

| **Cell Type** | **Cell surface marker definition** |
| --- | --- |
| Hematopoietic stem cells (HSC) | Lin- Sca1+ Kit+ CD150+ CD48- |
| Multipotential progenitor cells (MPP) | Lin- Sca1+ Kit+ CD150- CD48lo/- |
| Restricted hematopoietic progenitor cells 1 (HPC1) | Lin- Sca1+ Kit+ CD150- CD48+ |
| Restricted hematopoietic progenitor cells 2 (HPC2) | Lin- Sca1+ Kit+ CD150+ CD48+ |
|  |  |
| Common myeloid progenitor cells (CMP) | Lin- Sca1- Kit+ CD34+ FcgRII/III(CD16/32)- |
| Granulocyte-macrophage progenitor cells (GMP) | Lin- Sca1- Kit+ CD34+ FcgRII/III(CD16/32)+ |
| Megakaryocyte progenitor cells (MkP) | Lin- Sca1- Kit+ CD150+ CD41+ |
| Pre-colony-forming-erythroid cells (PreCFU-E) | Lin- Sca1- Kit+ CD150+ Endoglin(CD105)hi FcγRII/III(CD16/32)- |
| Colony-forming-erythroid cells (CFU-E) | Lin- Sca1- Kit+ CD150- Endoglin(CD105)hi FcγRII/III(CD16/32)- |
|  |  |
| B lymphoid cells - Fig3E | B220+ |
| B lymphoid cells - Sup Fig 4D | B220+ and/or CD19+ |
| T cells | CD4+ and/or CD8+ |
| Myeloid | Mac1+ and/or Gr1+ and/or Ly6G+ and/or F4/80+ |
| Erythrocytes | Ter119+ |
| Natural killer (NK) cells | Nk1.1+ |
| Megakaryocytes | CD41+ |
|  |  |
| Lineage (Lin) markers | CD2, CD4, CD8, Gr1, F4/80, Ly6G, CD19, B220,Ter119, Nk1.1 |

**Supplementary Table 2. Blood cell numbers in *Mpl*-mutant mice at 8 weeks of age**

|  | **WBC**  **(x10^6^/ml)** | **RBC**  **(x10^9^/ml)** | **Platelets**  **(x10^6^/ml)** | **Neutrophils**  **(x10^6^/ml)** | **Lymphocytes**  **(x10^6^/ml)** | **Monocytes**  **(x10^6^/ml)** | **Eosinophils**  **(x10^6^/ml)** |
| --- | --- | --- | --- | --- | --- | --- | --- |
|  |  |  |  |  |  |  |  |
| ***C57BL/6* (n=33)** | 7.9 ± 2.4 | 10.3 ± 0.7 | 1248 ± 240 | 0.61 ± 0.58 | 6.8 ± 2.2 | 0.16 ± 0.09 | 0.12 ± 0.06 |
| *Mpl^-/-^*(n=22) | 7.7 ± 1.6 | 10.0 ± 0.6 | 133 ± 54 ^****^ | 0.58 ± 0.38 | 6.7 ± 1. 5 | 0.14 ± 0.05 | 0.08 ± 0.05 |
| *Mpl^TM/TM^* (n=31) | 7.3 ± 2.1 | 9.2 ± 1.0 ^****^ | 82 ± 44 ^****^ | 0.45 ± 0.19 | 6.3 ± 1.8 | 0.12 ± 0.07 | 0.11 ± 0.06 |
| *Mpl^IC36/IC36^* (n=25) | 8.5 ± 2.6 | 9.1 ± 0.9 ^****^ | 87 ± 43 ^****^ | 0.73 ± 0.55 | 7.1 ± 2.3 | 0.15 ± 0.10 | 0.09 ± 0.05 |
| *Mpl^Y565F/Y565F^* (n=15) | 7.3 ± 1.9 | 10.3 ± 1.0 | 1910 ± 260 ^****^ | 0.67 ± 0.56 | 6.0 ± 1.8 | 0.19 ± 0.10 | 0.11 ± 0.08 |
| *Mpl^Y599F/Y599F^* (n=20) | 7.9 ± 1.7 | 10.2 ± 0.8 | 916 ± 222 ^****^ | 0.60 ± 0.37 | 6.8 ± 1.6 | 0.20 ± 0.06 | 0.12 ± 0.10 |
| *Mpl^Y604F/Y604F^*(n=14) | 7.6 ± 1.9 | 10.3 ± 0.8 | 1456 ± 198 ^**^ | 0.69 ± 0.34 | 6.4 ± 1.7 | 0.16 ± 0.07 | 0.10 ± 0.05 |
|  |  |  |  |  |  |  |  |
| *C57BL/6* (n=37) | 7.0 ± 1.7 ^††††^ | 10.6 ± 2.2 ^††††^ | 1230 ± 262 ^††††^ | 0.48 ± 0.54 ^††††^ | 6.1 ± 1.4 ^††††^ | 0.14 ± 0.07 ^††††^ | 0.12 ± 0.08 |
| ***Jak2^V617F/+^ Mpl^+/+^* (n=15)** | 17.4 ± 5.6 | 18.3 ± 2.1 | 2069 ± 668 | 1.97 ± 0.76 | 14.3 ± 4.7 | 0.49 ± 0.30 | 0.25 ± 0.35 |
| *Jak2^V617F/+^ Mpl^-/-^* (n=12) | 16.1 ± 2.3 | 20.9 ± 1.3 ^†^ | 243 ± 61 ^††††^ | 1.45 ± 0.49 | 13.9 ± 2.0 | 0.27 ± 0.09 ^††^ | 0.08 ± 0.04 |
| *Jak2^V617F/+^ Mpl^TM/TM^* (n=18) | 15.0 ± 2.5 | 20.1 ± 1.5 | 218 ± 118 ^††††^ | 1.12 ± 0.34 ^††^ | 13.2 ± 2.1 | 0.23 ± 0.09 ^††††^ | 0.07 ± 0.04 |
| *Jak2^V617F/+^ Mpl^IC36/IC36^* (n=18) | 14.8 ± 3.4 | 19.5 ± 1.9 | 199 ± 65 ^††††^ | 1.05 ± 0.38 ^†††^ | 13.1 ± 3.0 | 0.22 ± 0.10 ^††††^ | 0.07 ± 0.05 |
| *Jak2^V617F/+^ Mpl^Y565F/Y565F^* (n=16) | 19.0 ± 2.9 | 16.8 ± 1.8 | 4189 ± 922 ^††††^ | 3.12 ± 0.67 ^††††^ | 14.6 ± 2.6 | 0.41 ± 0.17 | 0.21 ± 0.63 |
| *Jak2^V617F/+^ Mpl^Y599F/Y599F^* (n=18) | 15.8 ± 3.7 | 20.2 ± 2.6 | 853 ± 186 ^††††^ | 1.48 ± 0.51 | 13.6 ± 3.3 | 0.21 ± 0.09 ^††††^ | 0.10 ± 0.11 |
| *Jak2^V617F/+^ Mpl^Y604F/Y604F^* (n=16) | 22.9 ± 8.1 ^††^ | 20.7 ± 4.4 ^†^ | 3115 ± 558 ^††††^ | 2.93 ± 1.24 ^†††^ | 18.8 ± 6.6 ^††^ | 0.38 ± 0.26 | 0.08 ± 0.07 |
|  |  |  |  |  |  |  |  |
| *C57BL/6* (n=28) | 6.0 ± 2.0 | 10.3 ± 1.0 | 1203 ± 216 ^††††^ | 0.70 ± 0.67 | 5.0 ± 1.9 | 0.10 ± 0.06 | 0.09 ± 0.05 |
| *Rosa26^+/KI^* (n=12) | 5.8 ± 1.3 | 10.4 ± 0.8 | 1168 ± 310 ^†††^ | 0.87 ± 0.57 | 4.5 ± 1.1 | 0.13 ± 0.08 | 0.12 ± 0.16 |
| *VavCre^T/+^* (n=10) | 7.1 ± 2.3 | 9.6 ± 1.0 | 1076 ± 373 ^†††^ | 1.64 ± 1.65 | 4.9 ± 2.2 | 0.17 ± 0.10 | 0.10 ± 0.07 |
| ***hCalrdel52 Mpl^+/+^* (n=14)** | 7.7 ± 1.8 | 10.2 ± 0.6 | 1897 ± 421 | 1.03 ± 0.88 | 6.1 ± 1.6 | 0.14 ± 0.07 | 0.11 ± 0.08 |
| *hCalrdel52 Mpl^-/-^* (n=10) | 6.2 ± 1.8 | 10.4 ± 0.9 | 142 ± 53 ^††††^ | 0.51 ± 0.27 | 5.3 ± 1.7 | 0.10 ± 0.05 | 0.07 ± 0.05 |
| *hCalrdel52 Mpl^TM/TM^* (n=12) | 7.9 ± 1.3 | 9.6 ± 0.5 | 86 ± 48 ^††††^ | 0.68 ± 0.34 | 7.0 ± 1.3 | 0.11 ± 0.04 | 0.09 ± 0.03 |
| *hCalrdel52 Mpl^IC36/IC36^* (n=10) | 4.8 ± 1.8 ^††^ | 9.1 ± 0.7 ^†^ | 94 ± 36 ^††††^ | 0.77 ± 0.48 | 3.8 ± 1.6 ^††^ | 0.09 ± 0.04 | 0.06 ± 0.02 |
| *hCalrdel52 Mpl^Y565F/Y565F^* (n=10) | 8.0 ± 1.8 | 10.4 ± 0.6 | 2816 ±1269^††††^ | 0.84 ± 1.01 | 6.7 ± 1.7 | 0.14 ± 0.10 | 0.13 ± 0.09 |
| *hCalrdel52 Mpl^Y599F/Y599F^* (n=8) | 6.0 ± 3.0 | 10.3 ± 0.7 | 891 ± 152 ^††††^ | 0.90 ± 0.92 | 4.5 ± 1.9 | 0.11 ± 0.04 | 0.15 ± 0.10 |
| *hCalrdel52 Mpl^Y604F/Y604F^* (n=8) | 7.7 ± 1.0 | 11.0 ± 0.6 | 2107 ± 237 | 0.62 ± 0.23 | 6.6 ± 0.7 | 0.20 ± 0.12 | 0.09 ± 0.07 |

**P< 0.005, ****P< 0.0001 for comparison with C57BL/6 (top panel, bold) by by one-way ANOVA with Dunnett’s correction for multiple comparisons.

^†^P< 0.05, ^††^P< 0.005, ^†††^P< 0.001, ^††††^P< 0.0001 for comparison with *Mpl^+/+^;Jak2^V617F/+^* (middle panel, bold) or *Mpl^+/+^*;*hCalr-del52* (bottom panel, bold) by one-way ANOVA with Dunnett’s correction for multiple comparisons.

**Supplementary Table 3. Oligonucleotides used for CRISPR-generated mice**

|  | sgRNA1 | sgRNA2 | Donor Oligonucleotide |
| --- | --- | --- | --- |
| Mpl-TM | TGG GCC TAC TGC TGC TAA AG | GCT ATT GGC AGC AGC CCT GA | GTG ACT GCT CTG CTC CTG GTG CTG AGC CTC AGT GCC CTT CTG GGC CTA CTG CTG CTA AAG ***TGA*** AGG CAG TCC CCA TGC TAC TGC AGA CCT ATA CAT TCC TAC ACA CTA CCT TAT CCA TCC |
| Mpl-IC36 | GGG TGT ACT TAC AGG ACT TA | GCT ATT GGC AGC AGC CCT GA | CCC TCG CTT CCA GAC CTA CAC CGG GTC CTA GGC CAG TAC CTC AGA GAC ACT GCA GCC CTA ***TGA*** AGG CAG TCC CCA TGC TAC TGC AGA CCT ATA CAT TCC TAC ACA CTA CCT TAT CCA TCC |
| Mpl-Y565F | CAA CCT CAG ATG GAC TAC AG |  | CTC CCT AAG TCC TCA GAG AGC ACT CCT TTA CCT CTG TGT CCC TCC CAA CCT CAG ATG GAC T**tt** AGA GGA CTG CAA CCT TGC CTG CGG ACC ATG CCC CTG TCT GTG TGT CCA CCC ATG GCT GAG ACG GG |
| Mpl-Y599F | CCA ATA GCT TAG TGG TAG GT |  | CT GTG TGT CCA CCC ATG GCT GAG ACG GGG TCC TGC TGC ACC ACA CAC ATT GCC AAC CAC TC**a** T**t**C CTA CCA CTA AGC TAT TGG CAG CAG CCC TGA AGG CAG TCC CCA TGC TAC TGC AGA CCT ATA |
| Mpl-Y604F | CCT ACC TAC CAC TAA GCT AT |  | GCT GAG ACG GGG TCC TGC TGC ACC ACA CAC ATT GCC AAC CAC TCC TAC CTA CCA CTA AGC T**tc** TGG CAG CAG CCC TGA AGG CAG TCC CCA TGC TAC TGC AGA CCT ATA CAT TCC TAC ACA CTA CCT |
| Jak2-V617F | TGG TGT CTG TGT CTG TGG AG |  | GCA GCA AGC ATG ATG AGT CAG CTT TCT CAC AAG CAT TTG GTT TTG AAT TAT GGT GTC TGT **t**TC TGT GGA GA**a** GAG AGT AAG TAA AGC CAG CTG CTT GTC TTT GTC AAT GTC ATA GCC TGT CTC AGA ATC CTT |

STOP codons introduced into Mpl-TM and Mpl-IC36 mice are shown in italics; single codon mutations introduced into target loci are bolded in lower case.

**Supplementary Table 4. Antibodies used for cytometry and western analyses**

| **Antigen** | **Conjugate** | **Clone** | **Supplier** | **Catalog no.** |
| --- | --- | --- | --- | --- |
|  |  |  |  |  |
| **Western Blots** |  |  |  |  |
| Calr |  | D3E6 | Cell Signaling Technology | #12238 |
| Mutated Calr |  | CAL2 | Dianova | #DIA-CAL-250 |
| GFP |  | 7.1 and 13.1 | Roche | #11814460001 |
| Anti-Rabbit IgG | HRP |  | GE Healthcare | #LNA934V/AH |
| Anti-Mouse Ig | HRP |  | Southern Biotech | #1010-05 |
|  |  |  |  |  |
| **Fluorescence Cytometry** |  |  |  |  |
| B220 | A700, FITC | RA3-6B2 | In-house |  |
|  | PECy7 | RA3-6B2 | BD Bioscience | #552772 |
|  | BV650 | RA3-6B2 | BD Bioscience | #563893 |
| CD105 | PE | MJ7/18 | eBioscience | #12-1051-83 |
| CD117 (Kit) | BV711 | 2B8 | BD Bioscience | #563160 |
|  | EF710 | 2B8 | Invitrogen | #46-1171-80 |
| CD150 | BV421 | TC15-12F12.2 | Biolegend | #115926 |
|  | BV711 | TC15-12F12.2 | Biolegend | #115941 |
| CD16/32 | PerCP-Cy5.5 | 24G2 | BD Bioscience | #560540 |
| CD19 | A700, FITC | 1D3 | In-house |  |
|  | PE-Cy7 | 1D3 | BD Bioscience | **#552854** |
| CD2 | A700 | RM2-1 | In-house |  |
| CD34 | A647 | RAM34 | BD Bioscience | #560230 |
| CD4 | A700, PE, FITC | GK1.5 | In-house |  |
| CD41 | PE-Cy7 | MWReg30 | Biolegend | #133916 |
| CD45.2 | APC | S450-15-2 | In-house |  |
| CD45.1 | PE-Cy7 | A20 | BD Bioscience | #560578 |
| CD48 | FITC | HM48-1 | eBioscience | #11-0481-85 |
|  | APC-Cy7 | HM48-1 | BD Bioscience | #561242 |
| CD8 | A700, PE, FITC | 53-6-7 | In-house |  |
| F4/80 | A700, FITC | F4/80 | In-house |  |
| Gr1 | A700, FITC | RB6-8C5 | In-house |  |
| Ly6G | A700, FITC | 1A8 | In-house |  |
| Mac1 | A700, FITC | M170 | In-house |  |
| Mpl | Biotin | AMM2 | IBL | # 10403 |
| Nk1.1 | A700, FITC | PK126 | In-house |  |
|  | PerCP-Cy5.5 | PK126 | BD Bioscience | #551114 |
| Sca1 | A594 | E13 | In-house |  |
| Streptavidin | BV650 |  | BD Bioscience | #563855 |
| Ter119 | A700, FITC | Ter119 | In-house |  |
|  |  |  |  |  |
| **Mass Cytometry** |  |  |  |  |
| pAkt(S473) | 152Sm | D9E | Fluidigm | #3152005A |
| pMAPK1/3(T202/Y204) | 171Yb | D13.14.4E | Fluidigm | #3171010A |
| pSTAT5(Y694) | 147Sm | 47 | Fluidigm | #3147012A |
| CD48 | 176Yb | HM48-1 | Biolegend-Maxpar | #103433 |
| Kit | 141Pr | 2B8 | Biolegend-Maxpar | #105829 |
| CD150 | 143Nd | TC15-12F12.2 | Biolegend-Maxpar | #115933 |
| Sca1 | 149Sm | E13-161.7 | In-house |  |
| pJak2(Y1008) | 169Tm | D4A8 | Cell Signaling Technology | #8082BF |
| pSTAT3(Y705) | 156Gd | D3A7 XP R | Cell Signaling Technology | #9145BF |

A594, AlexaFluor594; A647, AlexaFluor647; A650, AlexaFluor650; A700, AlexaFluor700; BV650, Brilliant Violet-650; BV711, Brilliant Violet-711; FITC, fluorescein isothiocyanate; APC, Allophycocyanin; PE, phycoerythrin; PerCP-Cy5.5, peridinin chlorophyll protein-cyanine5.5 tandem, PE-Cy7, phycoerythrin-cyanine7 tandem, APC-Cy7, Allophycocyanin-cyanine7 tandem; Sm, Samarium; Yb, Ytterbium; Pr, Praseodymium; Nd, Neodymium; Tm, Thulium; Gd, Gadolinium.

**Supplementary Figures**

**Supplementary Figure 1**

(**A**) Median fluorescence intensity (MedFI) of anti-Mpl antibody staining on MkP (n=2-7) (**B**) Number of platelets (n=8-10), median fluorescence intensity (MedFI) of anti-Mpl antibody staining on (**C**) platelets (n= 8-10), (**D**) MkP (n= 5-7) and (**E**) HSC, MPP, HPC1 and HPC2 (n= 8-10) and (**F**) total number of HSC, MPP1, HPC1 and HPC2 per femur in wild-type C57BL/6, *Mpl^-/-^*, *Mpl^+/-^* and *Mpl^Y599F/Y599F^* mice (n=5-6). Each point represents data from an individual mouse and bars represent mean ± SD. ^††^ P<0.05; ^†††^ P<0.001 by unpaired two-tailed t test.

**Supplementary Figure 2.**

(**A**) *Mpl* RNA expression in LSK cells expressed as counts per million reads from RNA-seq analysis (n=2), (**B**) Number of CMP (Lin^-^Sca1^-^Kit^+^CD34^+^CD16/32^-^), GMP (Lin^-^Sca1^-^Kit^+^CD34^+^CD16/32^+^), preCFU-E (Lin^-^Sca1^-^Kit^+^ CD150^+^CD105^hi^CD16/32^-^) and CFU-E (Lin^-^Sca1^-^Kit^+^CD150^-^CD105^hi^CD16/32^-^) per femur in C57BL/6 and *Mpl-*mutant mice (n=11-38). (**C**) Number of B-cells (B220^+^ and/or CD19^+^), T-cells (CD4^+^ and/or CD8^+^), myeloid (CD11b^+^ and/or Ly6G^+^ and/or F4/80^+^ and/or Gr1^+^), erythroid (Ter119^+^) and natural killer cells (NK, Nk1.1^+^) in wild-type C57BL/6 and *Mpl*-mutant mice (n=11-37). (**D**) Number of total (left) and megakaryocytic colonies (right) derived from 2.5 x10^4^ C57BL/6 and *Mpl-*mutant whole BM cells in semi-solid cultures containing (**D**) SCF, Epo and IL3 or (**E**) IL3 and Tpo (n=3-6). Each point represents data from an individual mouse and bars represent mean ± SD. *< 0.05; **P< 0.005 for comparison with C57BL/6 by one-way ANOVA with Dunnett’s correction for multiple comparisons.

**Supplementary Figure 3.**

(**A**) Test contribution (% Ly5.2) to peripheral blood at the indicated timepoints after transplantation re-plotted from Fig. 3D for comparison by test *Mpl*-mutant (Ly5.2):competitor wild-type (C57BL/6, Ly5.1/2) ratio. n=3-12 recipient mice per donor cell mixture, per timepoint; triangles: 9:1 test:competitor ratio; squares, 1:1 and circles, 1:3) (**B**) Test contribution (% Ly5.2) to the indicated organs 20 weeks after transplantation in mice transplanted at 1:1 test:competitor ratio (n=3-12 recipients per donor cell mixture). B, blood; BM, bone marrow; S, spleen; LN, lymph node; T, thymus. Each point represents data from an individual mouse, bars represent mean + SD. *P< 0.05; **P< 0.005; ***P< 0.001, ****P< 0.0001 for comparison with C57BL/6-test transplanted recipients by one-way ANOVA with Dunnett’s correction for multiple comparisons.

**Supplementary Figure 4.**

(**A**) Mass cytometry profiles indicating amounts of phospho(p)Jak2, pStat3, pStat5, pMAPK1/3 and pAkt levels in unstimulated wild-type C57BL/6 HSC (Lin^-^Sca1^+^Kit^+^CD150^+^CD48^-^) and in C57BL/6 and *Mpl*-mutant HSC stimulated with Tpo (100 ng/ml) for 5 minutes. Results from one representative experiment are shown. **(B,C)** Detection of phospho(p)Jak2, pStat3, pStat5, pMAPK1/3 and pAkt by mass cytometry (expressed as arcsinh-transformed median intensity relative to unstimulated C57BL/6 cells) in C57BL/6 and *Mpl^Y565F/Y565F^* HSC (Lin^-^Sca1^+^Kit^+^CD150^+^CD48^-^, n=3) (**B**) stimulated with 100 ng/ml Tpo for 30, 60 or 120 minutes and (**C**) stimulated with 1, 10, 100 ng/ml Tpo for 5 minutes (n=2-3). Paired t-tests were performed for C57BL/6 and Mpl^Y565F/Y565F^-mutant cells at each timepoint (B) or Tpo-concentration (C); no significant differences were evident.

**Supplementary Figure 5.**

(**A**) Platelet and (**B**) red blood cell numbers in compound *Mpl*-mutant;*Jak2^V617F^* mice (n=12-18) compared to Mpl mutant mice (n= 14-31) at 8 weeks of age. (**C**) Platelet and (**D)** red blood cell numbers (n=8-12), in compound *Mpl-*mutant;*hCalr-del52* compared to Mpl mutant mice (n=14-31) at 8 weeks of age (n=8-26). Data in panels A and C are reproduced from Fig. 1D, Fig. 2C, Fig. 6A and Fig. 6D to allow direct comparison of each *Mpl* mutant with respective *Jak2^V617F/+^* and hCalr-del52 compound mutant. Each point represents data from an individual mouse, bars represent mean + SD. *P< 0.05; ****P< 0.0001 (one-way ANOVA with Tukey’s correction for multiple comparisons).

**Supplementary Figure 6.**

Representative H&E-stained histological sections of sternums from (**A**) *Mpl^+/+^;Jak2^V617F/+^*, *Mpl^Y565F/Y565F^*;*Jak2^V617F/+^* and *Mpl^Y599F/Y599F^*;*Jak2^V617F/+^* mice at 8 weeks of age and (**B**) *Mpl^+/+^;Calr-del52*, *Mpl^Y565F/Y565F^*;*Calr-del52* and *Mpl^Y599F/Y599F^*;*Calr-del52* mice at 8 and 40 weeks of age. Images were acquired at 20x magnification. Bar: 100 μm.

**Supplementary Figure 7.**

(**A**) Structure of the *Rosa26-hCALR-del52* locus before (+/KI) and after Cre-mediated recombination (*hCalr-del52*). (**B**) Flow cytometric measurement of GFP-expression in CD41^+^ platelets (left) and mononucleated BM cells (right) in selected examples of *hCalr-del52* mice relative to wild-type C57BL/6 negative control. (**C**) Immunoblot analysis of mutated (mut) and wild-type (wt) Calr and GFP in mononucleated BM cells sorted for GFP low or high expression from mice with indicated genotypes. Platelet numbers in individual mice analysed are also shown. (**D**) Correlation of platelet number and %GFP^hi^ platelets in *hCalr-del52* mice. Statistical significance was determined by Pearson correlation, r=0.9076, P< 0.0001, n=18.

**Supplementary Figure 8.**

Representative flow cytometry profiles of lineage negative BM cells to illustrate gating strategies for GMP, CMP, PreCFU-E, CFU-E, MkP, HPC-1, HPC-2, MPP and HSC (top) and from whole BM for B cells, T cells, NK cells, erythroid and myeloid cells (bottom). See supplementary Table 3 for cell surface marker definitions for each population.


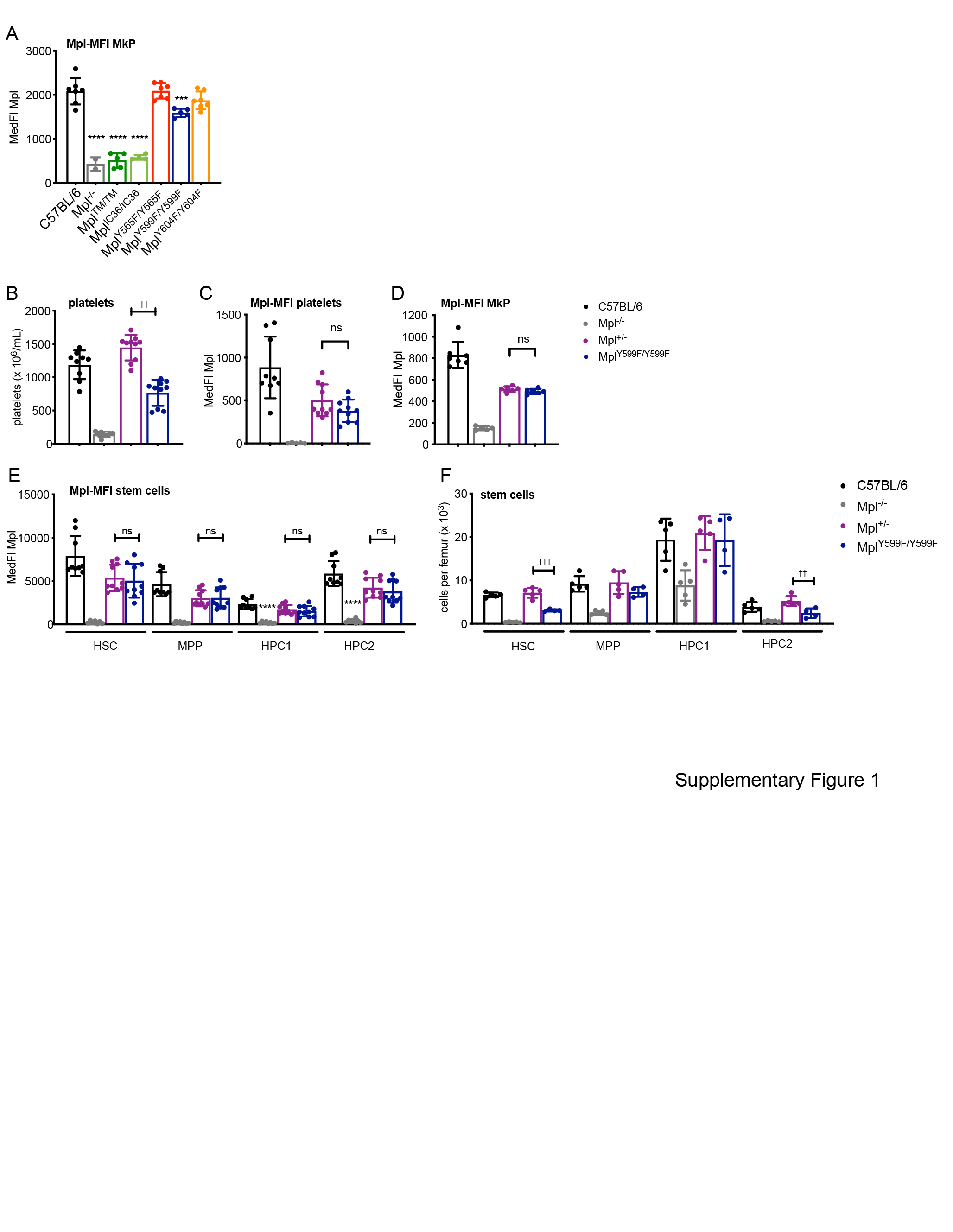
 **
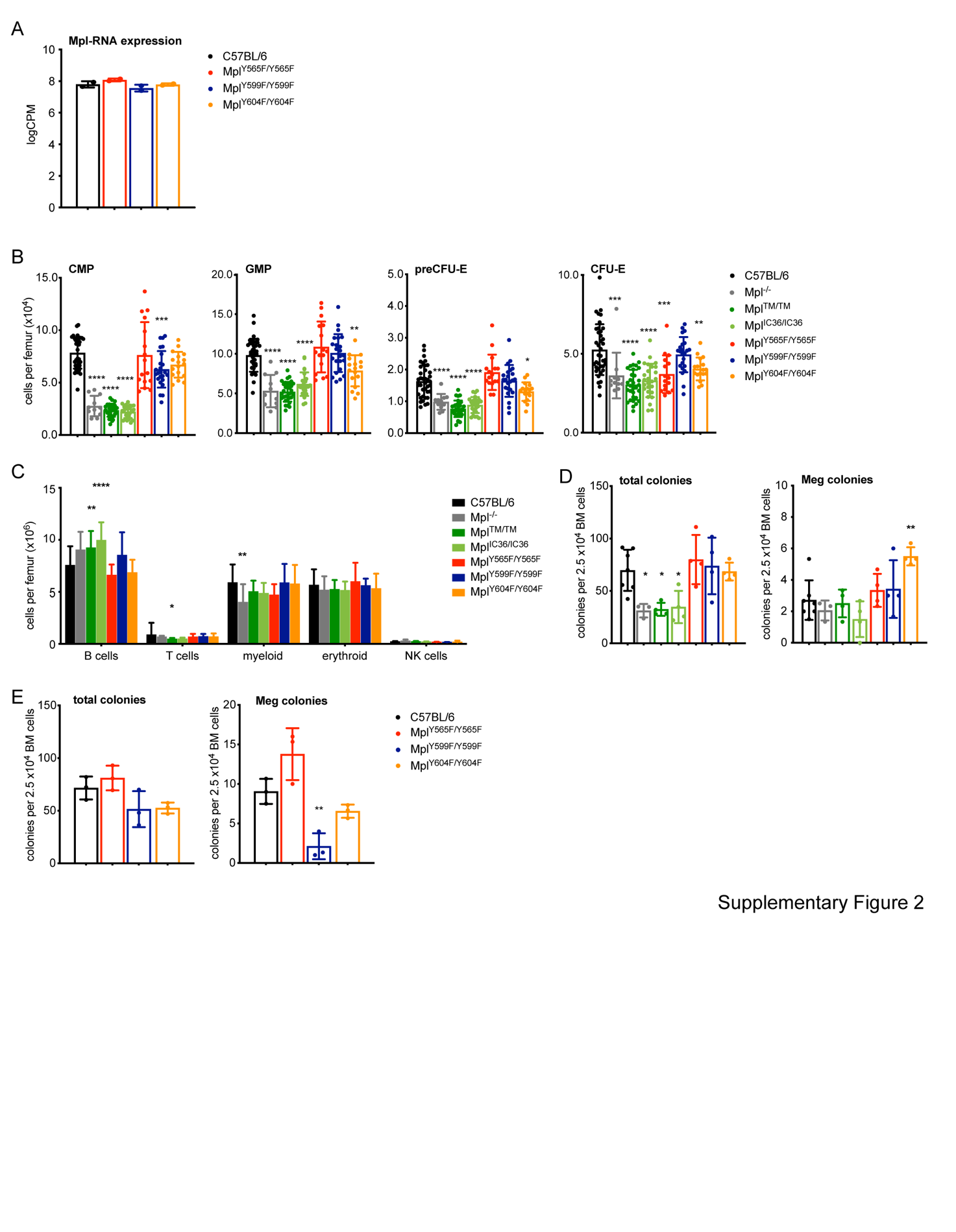
**


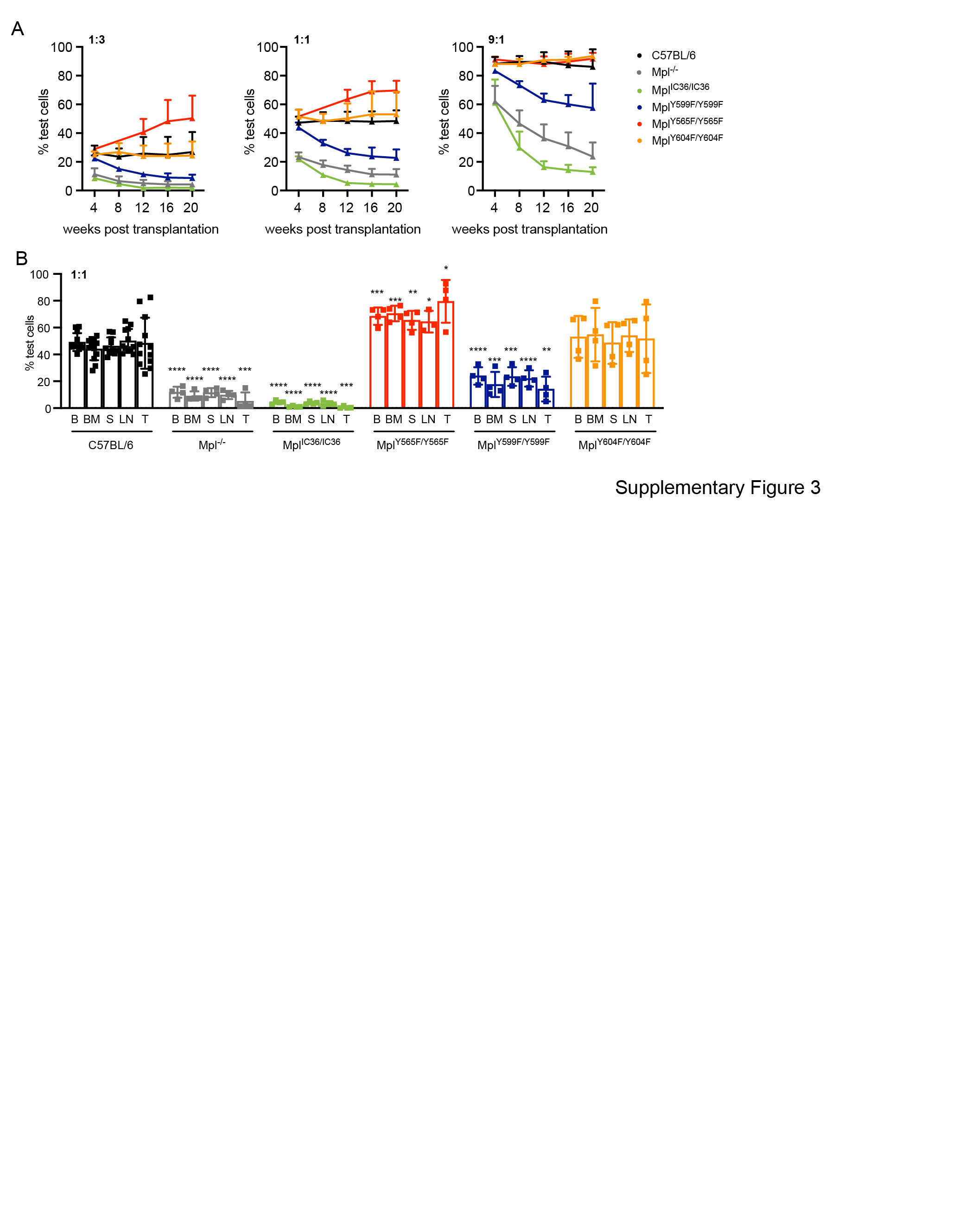

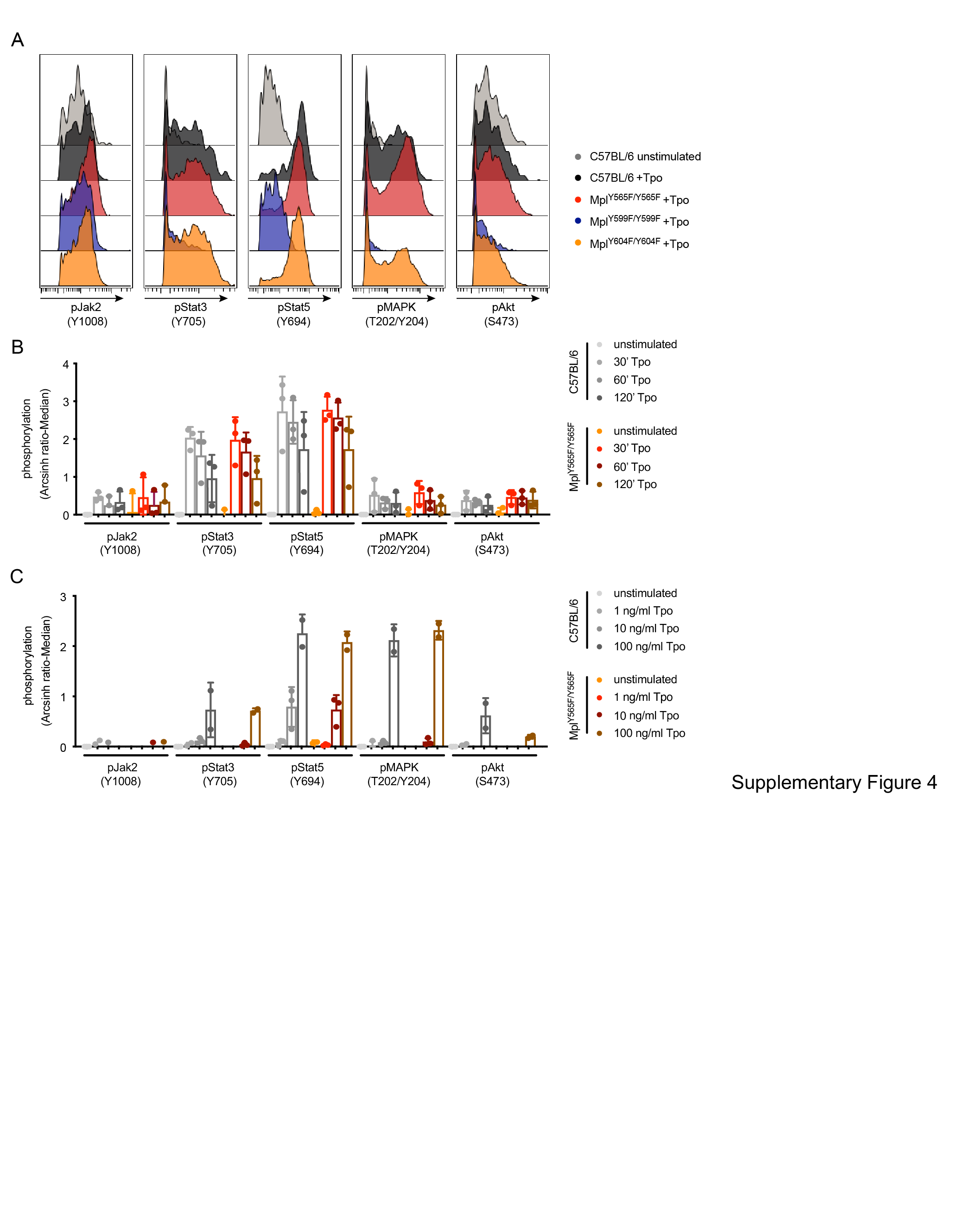


**
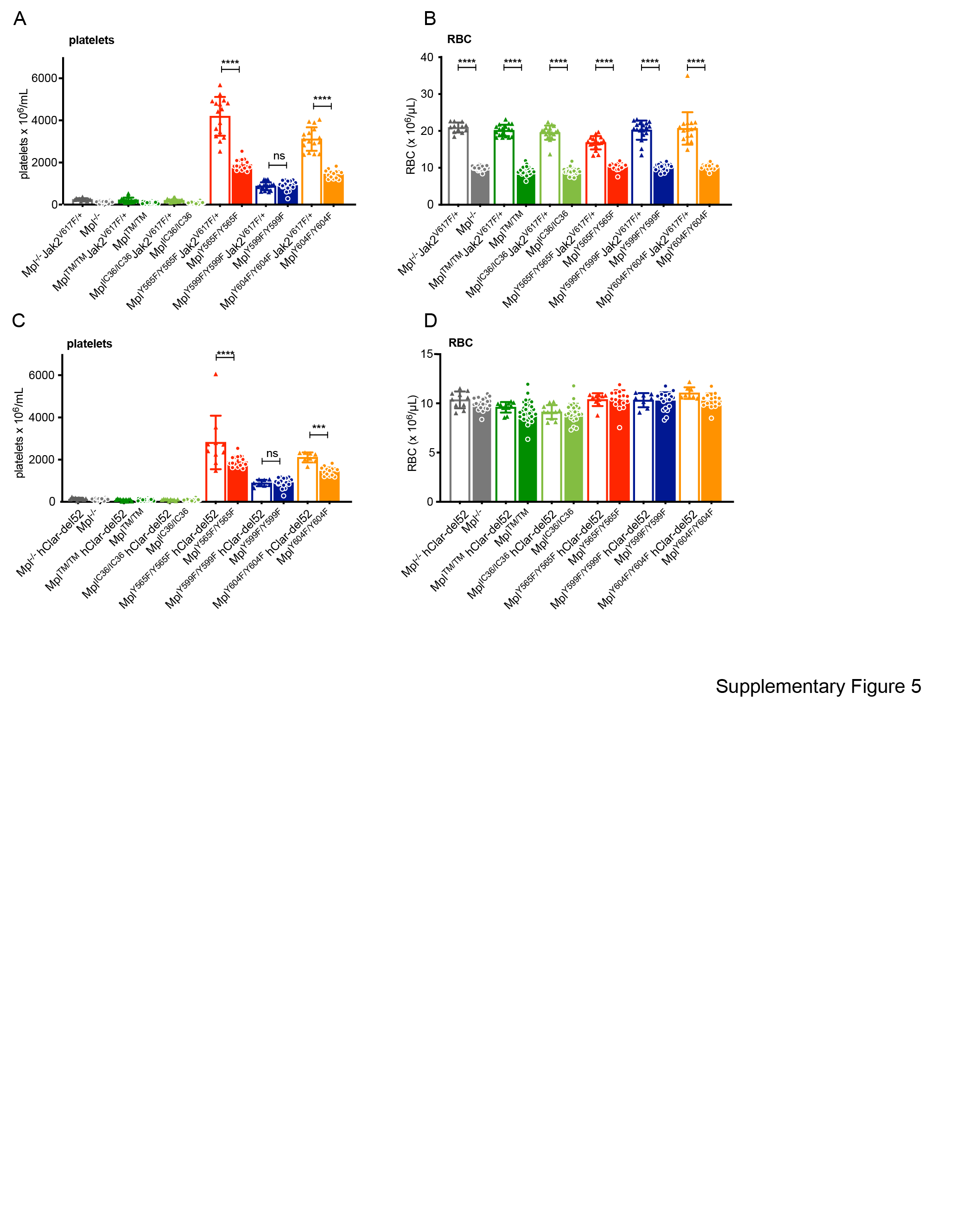
**


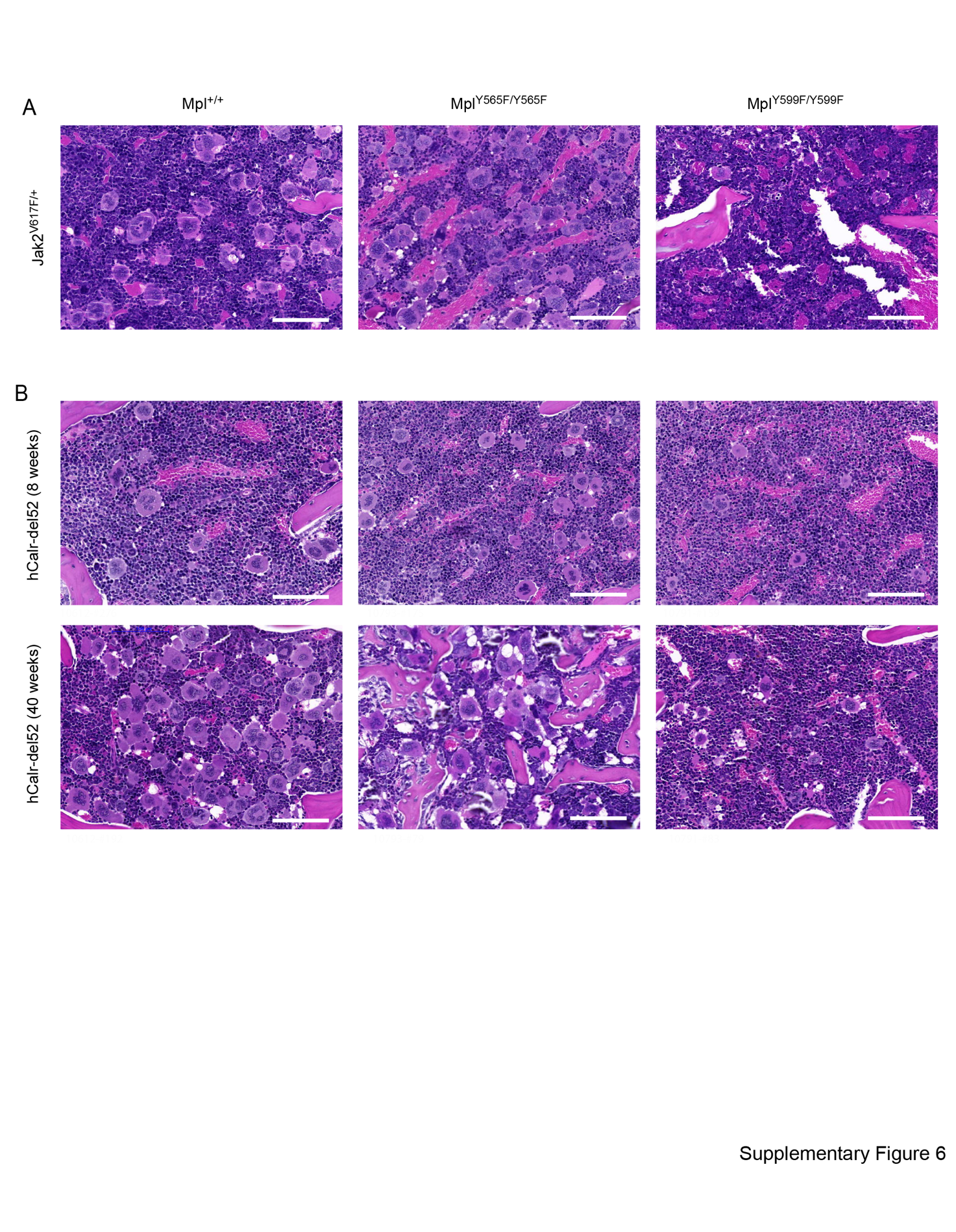


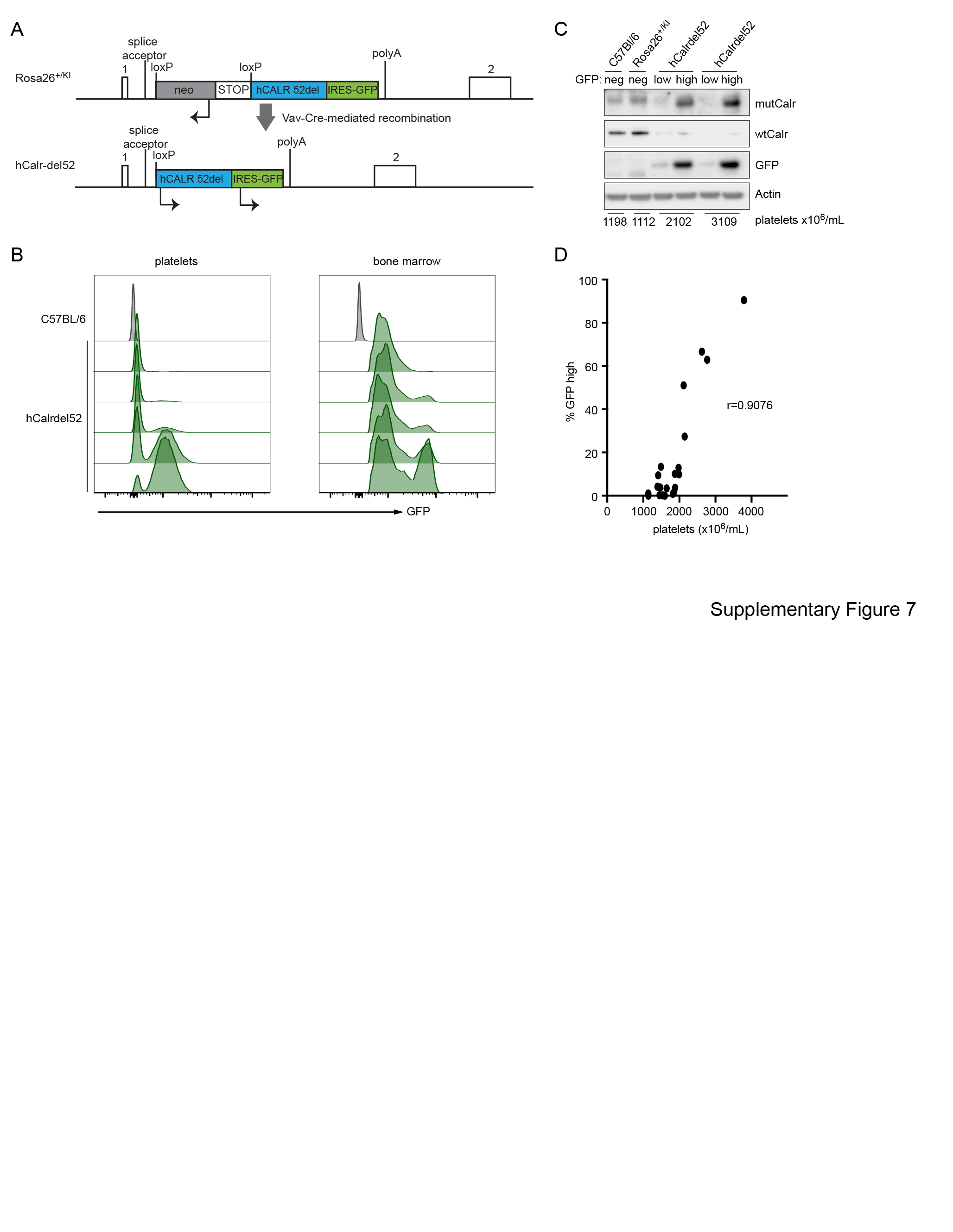


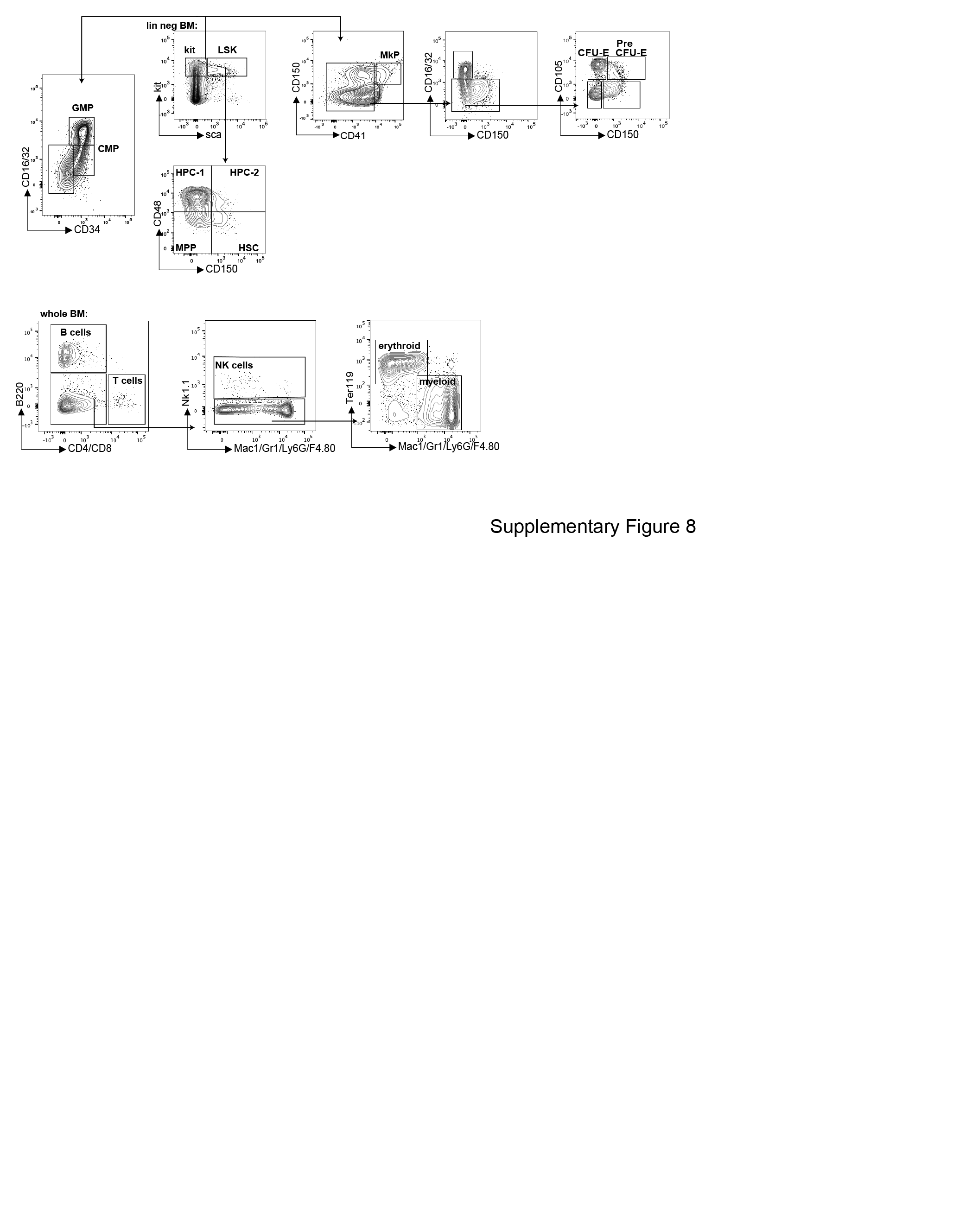

Supplement: Supplementary file 1 — Supplemental material [file 41375_2024_2219_MOESM1_ESM.docx]
